# Photo-cleavable purification/protection handle assisted synthesis of giant modified proteins with tandem repeats†
†Electronic supplementary information (ESI) available. See DOI: 10.1039/c9sc03693h


**DOI:** 10.1039/c9sc03693h

**Published:** 2019-08-12

**Authors:** Xueyi Liu, Jiazhi Liu, Zhichao Wu, Liangbiao Chen, Siyao Wang, Ping Wang

**Affiliations:** a Shanghai Key Laboratory for Molecular Engineering of Chiral Drugs , School of Chemistry and Chemical Engineering , Shanghai Jiao Tong University , 800 Dongchuan Road , Shanghai 200240 , P. R. China . Email: wangp1@sjtu.edu.cn; b Key Laboratory of Exploration and Utilization of Aquatic Genetic Resources , Ministry of Education , College of Fisheries and Life Science , Shanghai Ocean University , Shanghai 201306 , China

## Abstract

Proteins with tandem repeats have essential physical or biological roles in cells and have been widely investigated as biomaterials or vaccines. Chemically derived proteins with tandem repeats would be beneficial for research, owing to their accurate structures, possibly with precise modifications, produced by chemical synthesis. Traditional protein synthesis often leads to severe handling loss due to multiple ligations and HPLC purifications. To improve the protein synthesis efficiency, we developed a purification/protection handle consisting of a His_6_ tag and a photo-labile linker. This handle has great potential to facilitate purification with immobilized metal affinity chromatography techniques and also provides orthogonal protection for N-terminal Cys. The synthesis of the model proteins Muc1 and antifreeze glycoprotein mimics shows that the handle decreases the requirement for HPLC and enables both convergent and sequential assembly of peptide segments.

## Abstract 2

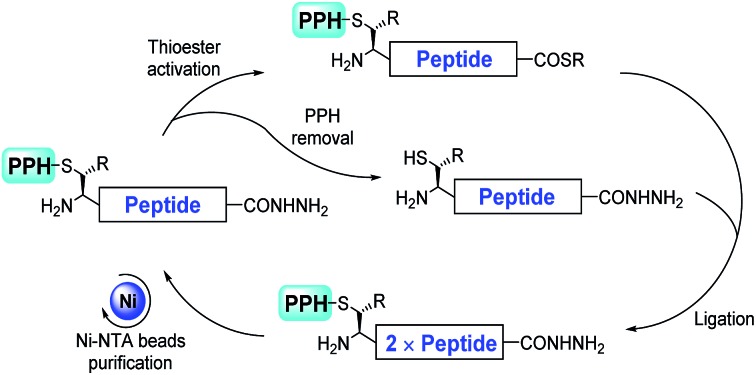
A new N-terminal protection/purification handle (PPH), which contained a His_6_ tag for purification and a photo-cleavable linker, facilitates the efficient synthesis of large proteins with tandem repeats.

## Introduction

Proteins with tandem repeats consisting of linear arrays of identical or similar repeating sequences have been shown to have important physical or biological functions.^1^–^3^ According to a genome analysis, about 30% of human proteins contain tandem repeats.^4^ For example, extensin,^5^ collagen,^6^ elastin^7^ and DSPP^8^ (dentin sialophosphoprotein) are essential structural proteins. Silk proteins from spiders and silkworms consist of tandem repeat sequences.^9^,^10^ Moreover, some proteins with tandem repeats have RNA or DNA binding ability,^11^*via* their repetition domain. For instance, the ‘RGG box’ is the key domain for RNA binding in proteins such as FMRP^12^ (fragile X mental retardation protein), and STPR^13^ (score and three amino acid peptide repeat) is a DNA binding domain with conserved repeat sequences in animals.

**Fig. 1:**
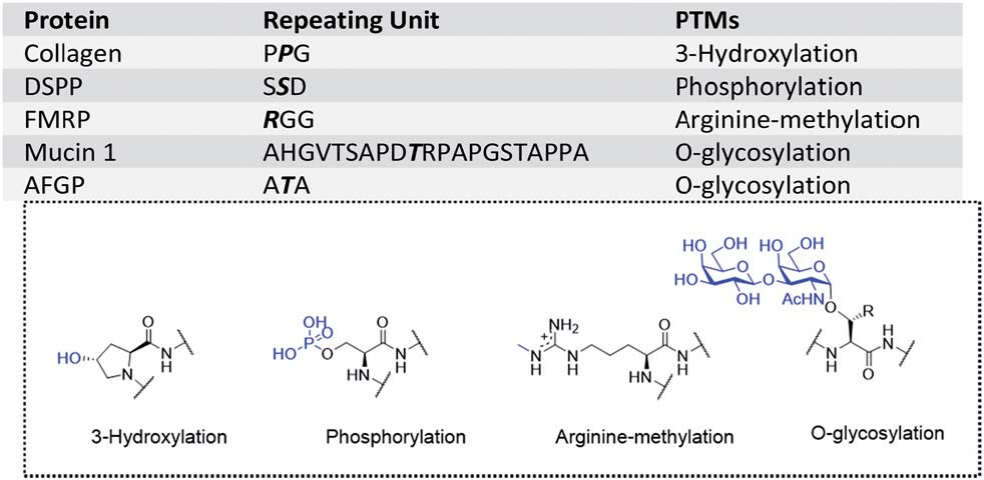
Examples of tandem repeats in proteins and the corresponding modifications (modified residues are highlighted in bold and italic letters). PTMs: post-translational modifications.

These tandem repeat domains are often post-translationally modified, and these modifications have key roles in their functions. For example, DSPP is phosphorylated on serine residues in its tandem repeat domain, thus enabling its calcium coordination.^14^ Glycosylated mucin^15^ and OGFR^16^ (opioid growth factor receptor) have been found to be immunogenic in cancer patients, and the repeating units of mucin have been widely studied as a cancer vaccine or diagnostic marker.^17^–^20^ AFGPs (antifreeze glycoproteins) in Antarctic and Arctic fishes provide protection against freezing,^21^ and consist of Alanine-Threonine-Alanine (ATA) as the smallest repeating unit, with a disaccharide (d-Galβ1-3-d-GalNAcα1) on each threonine residue (Fig. 1). The hydroxyl groups of the disaccharide are essential for the antifreeze activity.^22^,^23^ O-GalNAc modified AFGPs (monosaccharide AFGP mimics) have similar activities, whereas AGFPs without saccharides lack antifreeze activity.^24^–^26^

The biological and physical properties of proteins with tandem repeats might support their application as biomedicines and biomaterials in the future. However, proteins with PTMs are normally expressed as heterogeneous mixtures, thus restricting studies on the effects of PTMs. Chemical synthesis of proteins has been a successful strategy for obtaining homogeneously modified proteins.^27^–^29^ This strategy generally uses SPPS^30^,^31^ (solid phase peptide synthesis) to produce decorated peptide fragments, and then assembles these fragments into proteins through peptide ligation reactions.^32^–^34^ However, the handling loss during multi-step reactions and HPLC purifications has limited the synthetic application of large proteins.

Herein, we design a new N-terminal protection/purification handle (PPH), which facilitates the efficient synthesis of large proteins with tandem repeats by decreasing the number of required ligation/HPLC purification steps. To enable fewer HPLC purification steps, previous studies have used ligation on a solid phase,^35^–^39^ or affinity tag^40^ assisted purification. Among these strategies, we reasoned that the His_6_ tag^41^,^42^ would be well-suited for our purpose because (1) immobilized metal affinity chromatography purification for His_6_ tagged proteins is a well-established method, (2) common additives in native chemical ligation^34^ (NCL) reactions such as denaturing reagents are tolerated in this purification procedure, and (3) the loading and elution of the peptide can be achieved by adjusting the pH under mild conditions (pH 7.0 to 3.0).

**Fig. 2:**
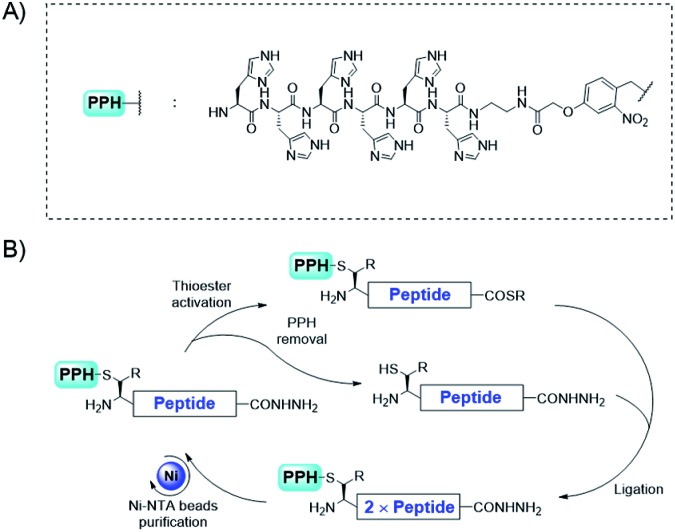
The convergent synthetic strategy for proteins with tandem repeats through purification/protection handle assistance.

To decrease the number of ligations, we used convergent synthesis. The N-terminal cysteines (or beta-thio amino acids) of peptides were required to be orthogonally protected (Fig. 2). Several orthogonal protection motifs were available for the N-terminal cysteine, but a photo-cleavable motif^43^,^44^ was chosen, owing to its reliable and fast deprotection under various conditions, thus making it more convenient than Thz^45^ (thiazolidine) and Acm^46^ (acetamidomethyl) protecting groups. For example, *ortho*-nitrobenzyl (*o*-NB) was used to protect cysteine and the deprotection was shown to be fast and efficient with UV irradiation.^47^ Also, this type of protection group has been successfully applied as an efficient cage/uncage system for cysteines in cells, suggesting its reliability under complex conditions. Moreover, the photo-cleavable motif could be further functionalized to incorporate a His_6_ tag to enable easy purification.

We demonstrated the applications of this special purification handle in the chemical synthesis of Muc1 (mucin 1) and a series of homogeneous AFGP mimics.^48^–^50^ One of them contained 240 amino acids (35 kDa), and is, to our knowledge, the largest chemically synthesized protein decorated with glycans reported to date. Furthermore, we evaluated the antifreeze properties of these synthetic AFGP mimics with thermal hysteresis (TH) analysis and examination of the dynamic ice crystal morphology.

## Results and discussion

### Design of the purification/protection handle

NCL^34^ has been shown to be the most efficient method for ligating unprotected peptides into proteins. This reaction is based on the thio-exchange between a peptide with an N-terminal cysteine (or another beta-thio amino acid) and another peptide with a C-terminal thioester and subsequent S to N acyl transfer to yield an amide bond. With or without further desulfurization, ligations can be achieved at sites with cysteine, alanine, or other residues if a beta-thio amino acid is used in the ligation step.^51^–^54^

For convergent assembly of the peptide, we designed our PPH to contain a His_6_ tag for purification and a photo-cleavable linker for orthogonal protection of the N-terminal cysteine (Fig. 2A). At the C-terminus, a thioester precursor is preferred because it would enable conversion to a thioester before ligations but remain inert when it is not needed. A C-terminal hydrazide was developed by Liu and co-workers,^55^,^56^ and its activation method developed by Dawson and co-workers^57^ can cleanly transform the hydrazide into a thioester; thus, we used C-terminal hydrazide as a thioester precursor (Fig. 2B).

### The synthesis of the photo-cleavable motif

**Fig. 3:**
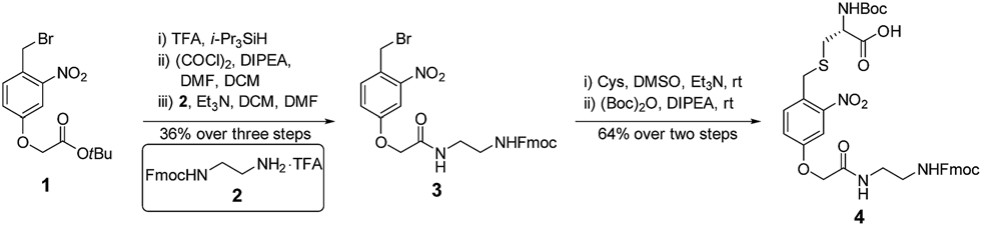
The synthesis of photo-cleavable building block **4**.

The synthesis of **4** started from the *tert*-butyl deprotection of compound **1**,^58^ followed by acyl chlorination and coupling with amine **2** 59 to afford compound **3** (36% yield over 3 steps). Benzyl bromide **3** reacted with the thiol group of cysteine, and this was followed by Boc protection of the α-amine to yield building block **4** (64% yield over 2 steps) (Fig. 3).

### Model peptide (Muc1)

**Fig. 4:**
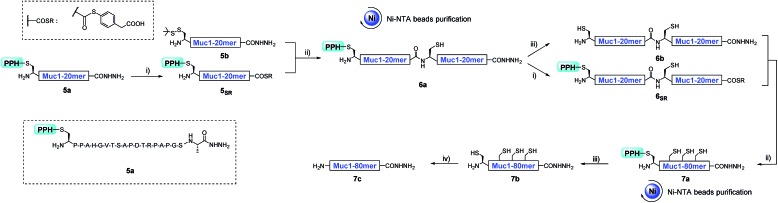
The synthesis process of **7c** with a convergent strategy. (i) 6 M Gnd·HCl, 0.2 M Na_2_HPO_4_, 0.2 M MPAA, acac, pH 3.0, rt, 4 h; (ii) 6 M Gnd·HCl, 0.2 M Na_2_HPO_4_, TCEP, pH 6.5, 37 °C, 1 h; (iii) 6 M Gnd·HCl, 0.2 M Na_2_HPO_4_, DTT, semicarbazide hydrochloride, pH 3.0, UV light (*λ* = 365 nm), rt, 15 min; (iv) 6 M Gnd·HCl, 0.2 M Na_2_HPO_4_, TCEP, GSH, VA-044, pH 7.0, 65 °C, 9 h (Gnd·HCl, guanidinium hydrochloride; MPAA, mercaptophenylacetic acid; TCEP, triscarboxyethylphosphine acid; GSH, glutathione; VA-044, 2,2′-azobis-[2-(2-imidazolin-2-yl)propane] dihydrochloride; DTT, dithiothreitol).

**Fig. 5:**
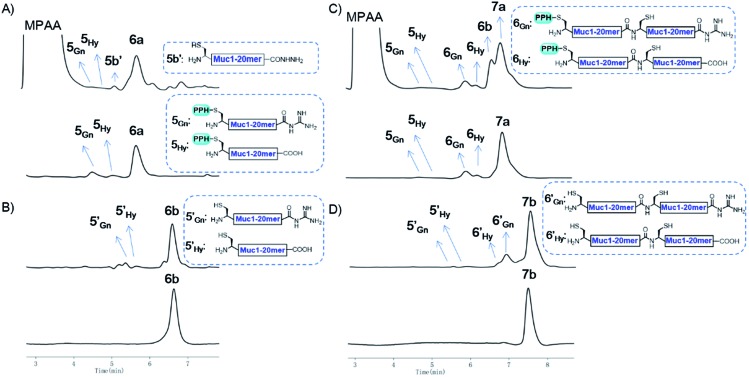
(A) Analytical HPLC results of crude **6a** (top) and Ni-NTA resin purified **6a** (bottom); (B) analytical HPLC results of crude **6b** (top) and HPLC purified **6b** (bottom); (C) analytical HPLC results of crude **7a** (top) and Ni-NTA resin purified **7a** (bottom); (D) analytical HPLC results of crude **7b** (top) and HPLC purified **7b** (bottom). HPLC conditions: detection at *λ* = 214 nm, gradients: 2–30% MeCN in H_2_O over 10 min.

As a proof of concept, we first used the PPH in the assembly of Muc1. Peptide segment **5a**, synthesized by Fmoc-SPPS, was transformed to the corresponding thioester **5_SR_** (see Fig. 4 and the ESI†). The following NCL reaction was initiated by adjustment of the pH to 6.5 and the addition of fragment **5b**, which was synthesized by Fmoc-SPPS as well (see the ESI†). This reaction reached completion in 1 h and yielded the ligation product **6a** according to LC-MS monitoring (Fig. 4 and 5A). We observed that trace amounts of byproducts due to hydrolysis (**5_Hy_**) and guanidination (**5_Gn_**) of the thioester were formed during activation of hydrazide, according to LC-MS, and the amounts of these two byproducts slightly increased in the ligation step (Fig. 5A). The ligation mixture was then loaded on pre-equilibrated Ni-NTA beads. The thiol additive and peptide contaminants without the His_6_ tag, *i.e.*, MPAA and **5b′**, were removed by washing the beads with phosphate buffer at pH 6.5. These impurities might coelute with the product if HPLC or other purification methods are used, and thus purification might be difficult with traditional methods. The **6a** on the beads was then eluted by washing with phosphate buffer at pH 3.0 (Fig. 4 and 5A).

With peptide hydrazide **6a**, PPH removal was accomplished with a previously reported method with minor modifications.^47^ Under weak acidic conditions (in the elution buffer, pH 3.0), the eluted **6a** was subjected to irradiation with UV light (365 nm) in the presence of semicarbazide as a scavenger and DTT as an antioxidant. The deprotection was highly efficient and was completed in 15 min to yield **6b** according to LC-MS monitoring. HPLC was used to purify **6b**, because the byproducts **5′_Gn_** and **5′_Hy_** could participate in the next round of NCL, despite their low abundance (Fig. 4 and 5B).

To obtain the peptide thioester, we used the 40 mer product **6a** in the elution for thioester activation without further purification, because the C-termini of byproducts **5_Gn_** and **5_Hy_** were deactivated and thus they could not participate in downstream reactions. Product **6a** in the elution buffer was concentrated to 1.8 mM *via* ultrafiltration and then subjected to thioester activation to yield **6_SR_** (see Fig. 4 and the ESI†). Then, thioester **6_SR_** was ligated with **6b** to yield **7a**, with a small amount of hydrolysis (**6_Hy_**) and guanidination (**6_Gn_**) of **6_SR_**. After PPH removal and HPLC purification, **7b** was obtained at 30% isolated yield starting from **5a**. After the subsequent desulfurization of Cys^54^ and HPLC purification, 80 mer Muc1 **7c** was obtained at 52% yield (see Fig. 4 and 5D and the ESI†).

### Synthesis of AFGP mimics

With the successful synthesis of the 80 mer Muc1, we then applied this strategy for the synthesis of larger *O*-glycosylated proteins, AFGP mimics. Fmoc-Thr(α-GalNAc(OAc)_3_)-OH, an essential glycosylated building block, was synthesized through the gold(i)-catalyzed glycosylation developed by Yu and co-workers.^60^–^63^ The peptide was synthesized with standard Fmoc-SPPS protocols. After completion of elongation, resin bound peptides were treated with 10% hydrazine monohydrate in DMF to remove the acetyl protecting group on GalNAc residues, and this was followed by acidolytic cleavage and HPLC purification to yield **8a** and **8b** (27% and 45% isolated yield, respectively, see the ESI†).

**Fig. 6:**
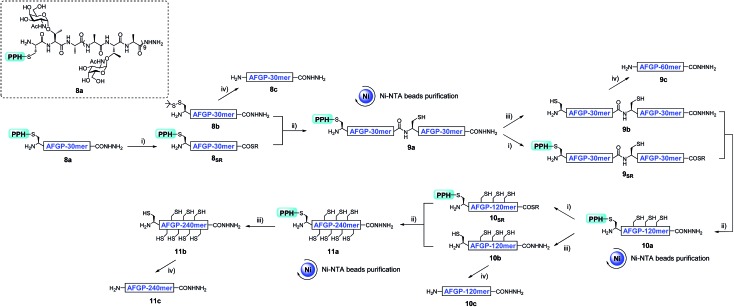
The synthesis process of AFGP mimics **8c**, **9c**, **10c**, and **11c** with a convergent strategy. (i) 6 M Gnd·HCl, 0.2 M Na_2_HPO_4_, 0.2 M MPAA, acac, pH 3.0, rt, 10 h; (ii) 6 M Gnd·HCl, 0.2 M Na_2_HPO_4_, TCEP, pH 6.5, 37 °C, 1 h; (iii) 6 M Gnd·HCl, 0.2 M Na_2_HPO_4_, DTT, semicarbazide hydrochloride, pH 3.0, UV light (*λ* = 365 nm), rt, 15 min; (iv) 6 M Gnd·HCl, 0.2 M Na_2_HPO_4_, TCEP, GSH, VA-044, pH 7.0, 65 °C, 9 h.

**Fig. 7:**
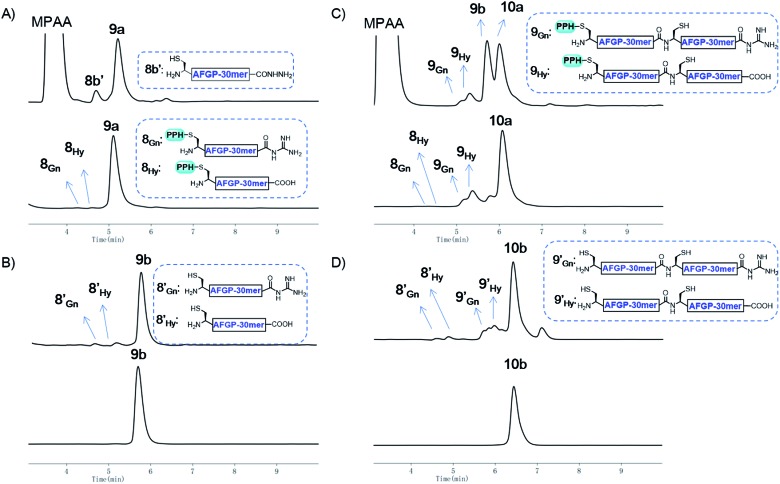
(A) Analytical HPLC results of crude **9a** (top) and Ni-NTA resin purified **9a** (bottom); (B) analytical HPLC results of crude **9b** (top) and HPLC purified **9b** (bottom); (C) analytical HPLC results of crude **10a** (top) and Ni-NTA resin purified **10a** (bottom); (D) analytical HPLC results of crude **10b** (top) and HPLC purified **10b** (bottom). HPLC conditions: detection at *λ* = 214 nm, gradients: 5–30% MeCN in H_2_O over 10 min.

The assembly of AFGP mimics followed the same procedure as Muc1 synthesis. As shown in Fig. 6 and 7A, **8a** was transformed to the thioester **8_SR_** and ligated with **8b** to yield **9a**, which was purified with Ni-NTA beads. Then, **9b** and **9_SR_** were obtained *via* PPH removal or thioester activation of **9a**, respectively (Fig. 6 and 7B). The following ligation of **9b** (with HPLC purification before use) and **9_SR_** (without HPLC purification) yielded **10a** (Fig. 6 and 7C), which was subjected to another ligation cycle to yield **11a**. After Ni-NTA purification, PPH removal and HPLC purification, **11b** was obtained (Fig. 6). Peptides **8b**, **9b**, **10b**, and **11b** were then desulfurized to yield AFGP mimics with different lengths: **8c**, **9c**, **10c**, and **11c**, respectively (see Fig. 6 and the ESI†).

**Fig. 8:**
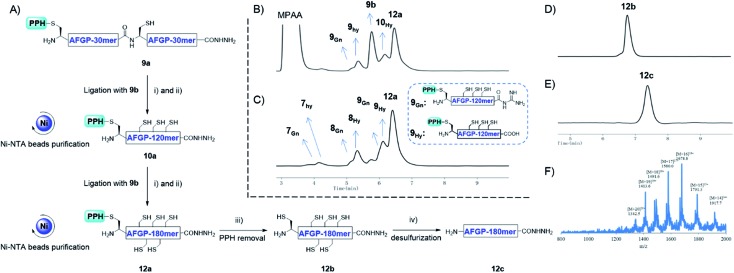
(A) The synthesis process of 180 mer AFGP mimic **12c** with a sequential strategy. (i) 6 M Gnd·HCl, 0.2 M Na_2_HPO_4_, 0.2 M MPAA, acac, pH 3.0, rt, 10 h; (ii) 6 M Gnd·HCl, 0.2 M Na_2_HPO_4_, TCEP, pH 6.5, 37 °C, 1 h; (iii) 6 M Gnd·HCl, 0.2 M Na_2_HPO_4_, DTT, semicarbazide hydrochloride, pH 3.0, UV light (*λ* = 365 nm), rt, 15 min; (iv) 6 M Gnd·HCl, 0.2 M Na_2_HPO_4_, TCEP, GSH, VA-044, pH 7.0, 65 °C, 9 h. (B) and (C) Analytical HPLC results of crude and Ni-NTA resin purified **12a**, respectively. (D) Analytical HPLC results of HPLC purified **12b**; (E) analytical HPLC results of HPLC purified **12c**; (F) ESI-MS results of **12c**.

In addition to using a convergent strategy, we applied this PPH in the sequential assembly of 60 mer peptides (obtained from a previous ligation) to yield a 180 mer product **12a**. Peptide hydrazide **9a** was activated to the thioester **9_SR_** and ligated with **9b** to obtain **10a**. After Ni-NTA bead purification the product **10a** was further activated to thioester **10_SR_**, and then ligated with another portion of **9b** to yield the 180 mer product **12a**, which was subjected to subsequent handle removal and desulfurization to yield a 180 mer AFGP mimic **12c** (Fig. 8A). The synthesis of the 180 mer AFPG mimic showed that sequential ligation can be achieved without HPLC purification before the final handle removal and desulfurization (Fig. 8). The sequential strategy also endowed AFGP with a flexible length, whereas the convergent method yielded AFGP only with 2^*n*^ peptide segments.

### CD spectra

**Fig. 9:**
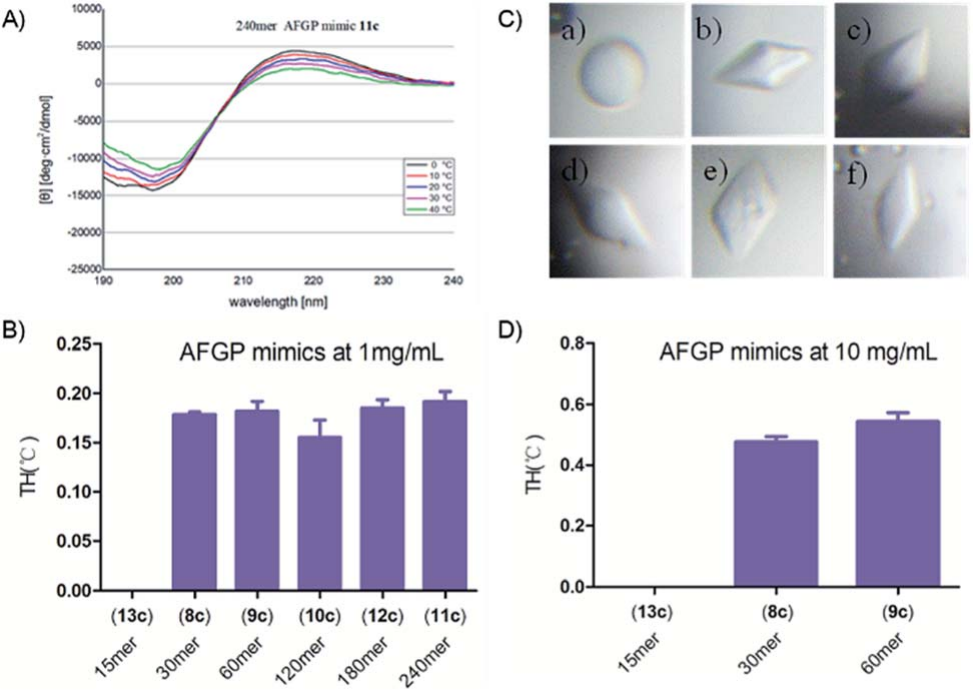
(A) CD spectra of the synthetic 240 mer AFGP mimic **11c** at temperatures ranging from 0 °C to 40 °C; (B) TH activity of the synthetic AFGP mimic at 1 mg mL^–1^; (C) ice crystal morphology in the presence of the synthetic AFGP mimic at 1 mg mL^–1^: (a) 15 mer AFGP mimic **13c**, (b) 30 mer AFGP mimic **8c**, (c) 60 mer AFGP mimic **9c**, (d) 120 mer AFGP mimic **10c**, (e) 180 mer AFGP mimic **12c**, and (f) 240 mer AFGP mimic **11c**; (D) TH activity of the synthetic AFGP mimics at 10 mg mL^–1^.

These synthetic AFGP mimics were then analysed by circular dichroism (CD) spectroscopy to confirm their secondary structures (see the ESI†). The spectra showed a positive maximum at 215 to 220 nm and a negative minimum at 190 to 200 nm (240 mer AFGP mimic as an example in Fig. 9A). With a temperature increase, these features were diminished, thus suggesting that the peptides adopted a PPII helix, a result consistent with previous characterization by Bush^64^*et al.*

### Evaluation of antifreeze activity

We next studied the antifreeze activity of these mimics with TH analysis and examination of the dynamic ice crystal morphology. According to previous studies,^48^,^49^ TH analysis of the monosaccharide AFGP mimics at a concentration of 10 mg mL^–1^ was performed. However, we found that monosaccharide AFPG mimics of 120 mer or greater were not fully dissolved at 10 mg mL^–1^. To compare the antifreeze activity of AFGP mimics with different lengths, we conducted TH tests with a protein concentration of 1 mg mL^–1^, at which all AFGPs were dissolved. The 15 mer mimic was previously found to be inactive^48^ and thus was used as a negative control in our study (see Fig. 9 and the ESI†). The shape of the ice crystal nuclei of the 15 mer solution at 1 mg mL^–1^ was spherical, whereas crystals in other AFPG mimic solutions formed hexagonal bipyramids (Fig. 9C), thus suggesting binding of AFGP mimics to the crystals. In addition, the gap between the melting point and freezing point, which is defined as the TH activity,^65^ was observed in solutions with mimics of 30 mer or longer lengths. However, these mimics showed similar TH activity at 1 mg mL^–1^ without any notable relationship with their length. We also determined the TH activity at a concentration of 10 mg mL^–1^ for the 30 mer and 60 mer AFGP mimics. With increasing concentrations, the TH activity increased to approximately 0.5 °C, whereas the 15 mer remained inactive (Fig. 9B and D).

## Discussion

Proteins with tandem repeats have been found to have many essential biological or physical roles in cells. Synthetically derived proteins with tandem repeats would provide homogeneous molecules for studying protein functions. Here, we developed a useful method using a removable purification/protection handle to achieve fast and efficient synthesis of proteins with tandem repeats. This method may facilitate the synthesis of more complex proteins with tandem repeats. The handle was efficiently removed by UV irradiation after ligation, even under denaturing conditions. The highly efficient purification and handle removal, resulting in less byproduct, were also beneficial for decreasing the unavoidable downstream HPLC purification. With Muc1 and monosaccharide AFGP mimics as models, we showed that the use of HPLC purification could be decreased, thus improving the efficiency. With this strategy, AFGP mimics with as many as 240 amino acids were obtained, thus representing the longest synthetic homogeneous AFGP mimics reported to date. We believe that the PPH strategy will be a valuable protocol for the synthesis of proteins with tandem repeats.

## Conflicts of interest

There are no conflicts to declare.

## Supplementary Material

Supplementary information
