# Supplementary material for: Photo-cleavable purification/protection handle assisted synthesis of giant modified proteins with tandem repeats
Source: Chem Sci. 2019 Aug 12;10(37):8694–700. doi: 10.1039/c9sc03693h (PMC6849634; doi:10.1039/c9sc03693h)
Supplement: Supplementary file 1 [file SC-010-C9SC03693H-s001.pdf]

Electronic Supplementary Material (ESI) for Chemical Science.
This journal is © The Royal Society of Chemistry 2019

Supporting Information

A Photo-Cleavable Purification/Protection Handle Assisted Synthesis of Giant Modified Proteins with Tandem Repeats

Xueyi Liu, ‡ [a] Jiazhi Liu, ‡ [a] Zhichao Wu,[b] Liangbiao Chen,[b] Siyao Wang [a] and Ping Wang *[a]

[a] Shanghai Key Laboratory for Molecular Engineering of Chiral Drugs, School of Chemistry and Chemical Engineering, Shanghai Jiao Tong University, 800 Dongchuan Road, Shanghai 200240, P. R. China.

[b] Key Laboratory of Exploration and Utilization of Aquatic Genetic Resources, Ministry of Education, College of Fisheries and Life Science, Shanghai Ocean University, Shanghai 201306, China.

‡ *Contributed equally*

Table of Contents

1. General information 3

2. Synthesis of building blocks for SPPS 3

3. Solid-phase peptide synthesis (SPPS) 7

4. The assembly of peptide segments 12

5. Metal-free desulfurization 29

6. Circular dichroism spectropolarimetry 35

7. Evaluation of antifreeze activity 36

8. NMR-spectra of novel compounds 37

9. High-resolution mass spectra of AFGP mimic **10c**, **12c**, **11c** 42

10. Reference 44

1.General information

1.1 Materials and instruments

All chemicals were obtained from commercial sources: Adamas, Macklin, Energy Chemicals, Sigma Aldrich, and were used without further purification unless otherwise stated. Amino acids, HATU, HOAt and 2-Cl(Trt)-Cl resins were acquired from GL Biochem (Shanghai). Ni-NTA resins were acquired from Smart-Lifesciences. Dry solvents were acquired from solvent purification system (Vigor YJC-7). Reactions were monitored by TLC plates and visualizations were performed with UV light (254 nm). UV irradiation was performed under UV Lamps from Gongben company (20 watt, LED, 365 nm). The flash chromatography was carried out on Teledyne ISCO CombiFlash Rf+. ^{1}H NMR spectra were recorded on Bruker AVANCE III HD 400 MHz; ^{13}C NMR spectra were recorded on Bruker AVANCE III HD 101 and 126 MHz. HRMS was recorded on Waters 2D H class & VION IMS QTOF MS.

Analytical LC-MS: Shimadzu LC-MS 2020; Column: ReproSil-Pur (C4/C18, 3 μm, 2.1 × 100 mm); Solvent A: 0.1% formic acid in water; Solvent B: 0.1% formic acid in acetonitrile.

Preparative reverse-phase HPLC: Shimadzu HPLC SPD-20A; Column: ReproSil (C4/C18, 5 μm, 10 × 150 mm); Solvent A: 0.05% TFA in water; Solvent B: 0.04% TFA in acetonitrile.

2. Synthesis of building blocks for SPPS

2.1 Synthesis of the photo-cleavable moiety of PPH

Preparation of compound S1

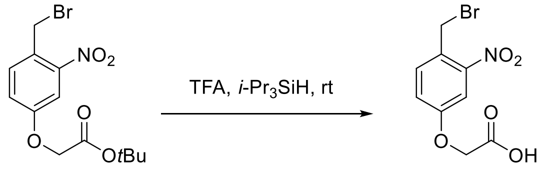

1 **S1**

To the solution of compound **1**[1] (2.3 g, 6.7 mmol, 1.0 equiv.) in TFA (2 mL), triisopropylsilane (*i*-Pr_3SiH) (20 μL) was added at 0 °C. Then the solution was stirred at room temperature for 3 h. After concentrated to ~200 μL with a nitrogen flow, the residue was precipitated with ice-cold Et_2O. The white solid was filtered and the residue solvent was removed *in vacuo* to afford compound **S1** as a white powder (1.5 g, 5.2 mmol, 77%). ^{1}H NMR (400 MHz, DMSO-d_6) δ 7.67 (d, J = 8.7 Hz, 1H), 7.58 (d, J = 2.7 Hz, 1H), 7.32 (dd, J = 8.6, 2.8 Hz, 1H), 4.89 (s, 2H), 4.86 (s, 2H). ^{13}C NMR (126 MHz, DMSO-d_6) δ 170.04, 158.73, 149.09, 134.40, 124.96, 120.52, 111.67, 65.37, 30.36. HRMS (ESI) calculated for $C_9H_8BrNO_5$: $[M-H]^-$ m/z 287.9508, Found: 287.9505.

Preparation of compound 3

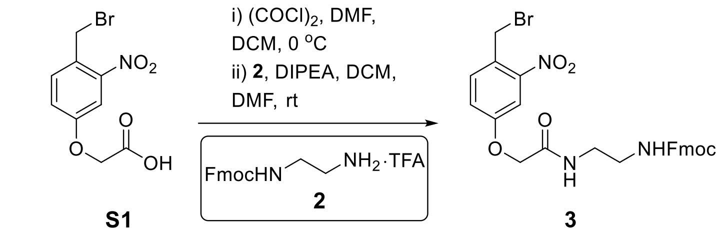

To the solution of compound **S1** (1.5 g, 5.2 mmol, 1.0 equiv.) in dry DCM (20 mL), oxalyl chloride (2 mL, 26.0 mmol, 5.0 equiv.) was added. After the addition of DMF (5 mL), the mixture was stirred at 0 °C for 30 min, and then the solvent was removed *in vacuo* to afford the crude compound as yellow oil. The crude product was dissolved in dry DCM (15 mL), and DIPEA (893 µL, 5.2 mmol, 1.0 equiv.) was added subsequently. Then the solution of trifluoroacetate salt **2**[2] (2.9 g, 10.4 mmol, 2.0 equiv.) in dry DMF (15 mL) was added. After 1 h, the solution was diluted with DCM, washed with water and brine. The organic phase was combined and dried with anhydrous Na_2SO_4. After filtration and concentration under reduced pressure, the residue was purified by flash chromatography to afford compound **3** as a white powder (1.4 g, 2.5 mmol, 47% over two steps). ^{1}H NMR (400 MHz, DMSO-d_6) δ 8.27 (d, J = 6.8 Hz, 1H), 7.89 (d, J = 7.5 Hz, 2H), 7.73 - 7.60 (m, 4H), 7.45 - 7.29 (m, 6H), 4.97 (s, 2H), 4.62 (s, 2H), 4.31 (d, J = 6.9 Hz, 2H), 4.21 (t, J = 6.9 Hz, 1H), 3.23 - 3.16 (m, 2H), 3.10 (t, J = 6.2 Hz, 2H). ^{13}C NMR (101 MHz, DMSO-d_6) δ 167.49, 158.63, 156.74, 149.03, 144.34, 141.19, 134.00, 128.06, 127.51, 125.58, 124.68, 120.57, 120.40, 112.16, 67.73, 65.81, 47.18, 42.93, 38.98, 30.41. HRMS (ESI) calculated for $C_{26}H_{24}BrN_3O_6$ $[M+H]^+$ 554.0942, Found: 554.0927.

Preparation of compound 4

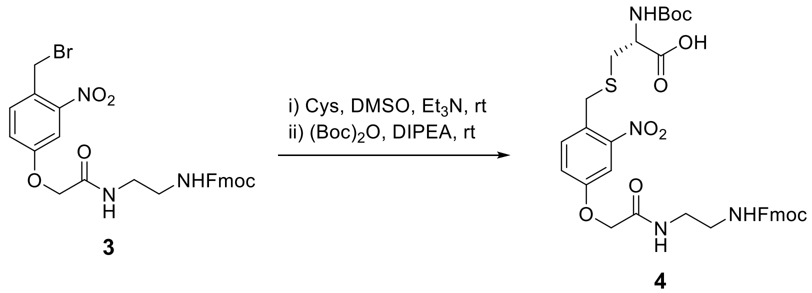

To the solution of compound **3** (1.4 g, 2.5 mmol, 1.0 equiv.) in DMSO (20 mL), NH_2-Cys-COOH (600 mg, 5.0 mmol, 2.0 equiv.) and Et_3N (1 mL, 7.4 mmol, 3.0 equiv.) were added. The mixture was stirred vigorously for 2.5 h, followed by the addition of $(Boc)_2O$ (2 mL, 15.0 mmol, 6.0 equiv.) and DIPEA (1 mL, 7.5 mmol, 3.0 equiv.). Then, the mixture was stirred at room temperature for another 3.5 h. The solution was directly purified by flash reverse phase chromatography. After lyophilization, compound **4** was obtained as a white powder (1.1 g, 1.6 mmol, 64% over 2 steps). ^{1}H NMR (400 MHz, DMSO-d_6) δ 8.23 (t, J = 5.8 Hz, 1H), 7.89 (d, J = 7.5 Hz, 2H), 7.68 (d, J = 7.4 Hz, 2H), 7.60 (d, J = 2.6 Hz, 1H), 7.51 (d, J = 8.6 Hz, 1H), 7.47 - 7.23 (m, 6H), 7.12 (d, J = 8.4 Hz, 1H), 4.58 (s, 2H), 4.31 (d, J = 6.9 Hz, 2H), 4.21 (t, J = 6.9 Hz, 1H), 4.11 - 3.93 (m, 3H), 3.21 (q, J = 6.4 Hz, 2H), 3.10 (q, J = 6.3 Hz, 2H), 2.78 (dd, J = 13.6, 4.8 Hz, 1H), 2.64 (dd, J = 13.6, 9.4 Hz, 1H), 1.39 (s, 9H). ^{13}C NMR (101 MHz, DMSO-d_6) δ 172.33, 167.11, 156.97, 156.23, 155.31, 148.53, 143.84, 140.68, 133.06, 127.55, 127.00, 125.91, 125.08, 120.07, 119.55, 111.67, 78.19, 67.23, 65.31, 53.30, 46.68, 38.46, 32.71, 32.11, 28.13,

26.84. HRMS (ESI) calculated for $C_{34}H_{38}N_4O_{10}S$: $[M-H]^-$ 693.2271, Found: 693.2256.

2.2 Synthesis of Fmoc-Thr(α-GalNAc)-OH

Preparation of compound S4

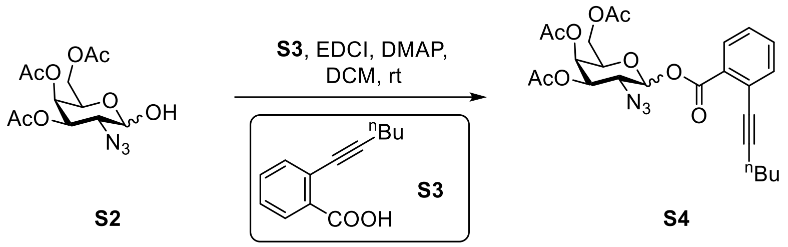

To the solution of **S2**[3] (7.8 g, 23.5 mmol, 1.0 equiv.) and **S3**[4] (7.4 g, 30.6 mmol, 1.3 equiv.) in dry DCM (150 mL), EDCI (9.0 g, 47.0 mmol, 2.0 equiv.) and DMAP (584 mg, 4.7 mmol, 0.2 equiv.) were added. Then the solution was stirred at room temperature for 4 h. Subsequently, the solution was diluted with DCM and washed with saturated $NaHCO_3$ solution twice. And then, the organic phase was concentrated under reduced pressure and purified by flash chromatography to afford compound **S4** as yellow oil (10.9 g, 21.0 mmol, α/β = 1:5, 89%).

S4α: ^{1}H NMR (400 MHz, Chloroform-*d*) δ 7.96 (d, *J* = 8.0 Hz, 1H), 7.56 (d, *J* = 7.8 Hz, 1H), 7.52 - 7.45 (m, 1H), 7.36 (t, *J* = 7.6 Hz, 1H), 6.60 (d, *J* = 3.6 Hz, 1H), 5.53 (d, *J* = 3.4 Hz, 1H), 5.42 (dd, *J* = 10.9, 3.2 Hz, 1H), 4.47 (t, *J* = 6.8 Hz, 1H), 4.16 (dd, *J* = 11.3, 7.3 Hz, 1H), 4.13 - 4.01 (m, 2H), 2.59 - 2.46 (m, 2H), 2.18 (s, 3H), 2.07 (s, 3H), 2.00 (s, 3H), 1.62 (s, 2H), 1.51 - 1.46 (m, 2H), 0.95 (t, *J* = 7.3 Hz, 3H). ^{13}C NMR (101 MHz, Chloroform-*d*) δ 170.26, 170.06, 169.78, 164.00, 135.19, 132.52, 130.87, 129.68, 127.40, 125.24, 97.13, 91.05, 79.76, 69.40, 69.04, 66.86, 61.04, 57.19, 30.72, 22.10, 20.67, 20.65, 20.62, 19.49, 13.67. HRMS (ESI) calculated for $C_{25}H_{29}N_3O_9Na$: $[M+Na]^+$ 538.1802, found 538.1816.

S4β: ^{1}H NMR (400 MHz, Chloroform-*d*) δ 8.02 (d, *J* = 8.0 Hz, 1H), 7.55 (d, *J* = 7.8 Hz, 1H), 7.51 - 7.44 (m, 1H), 7.37 - 7.31 (m, 1H), 5.79 (d, *J* = 8.5 Hz, 1H), 5.41 (d, *J* = 3.3 Hz, 1H), 4.96 (dd, *J* = 10.8, 3.3 Hz, 1H), 4.19 - 4.07 (m, 3H), 3.98 (dd, *J* = 10.8, 8.5 Hz, 1H), 2.50 (t, *J* = 7.1 Hz, 2H), 2.17 (s, 3H), 2.07 (s, 3H), 2.03 (s, 3H), 1.67 - 1.60 (m, 2H), 1.54 - 1.46 (m, 2H), 0.95 (t, *J* = 7.3 Hz, 3H). ^{13}C NMR (101 MHz, Chloroform-*d*) δ 170.35, 169.97, 169.65, 163.27, 134.72, 132.61, 130.64, 129.41, 127.18, 125.85, 97.34, 93.27, 79.01, 71.71, 71.49, 66.22, 60.89, 59.96, 30.66, 22.09, 20.66, 20.62, 20.60, 19.56, 13.67. HRMS (ESI) calculated for $C_{25}H_{29}N_3O_9Na$: $[M+Na]^+$ 538.1802, found 538.1816.

Preparation of compound S6

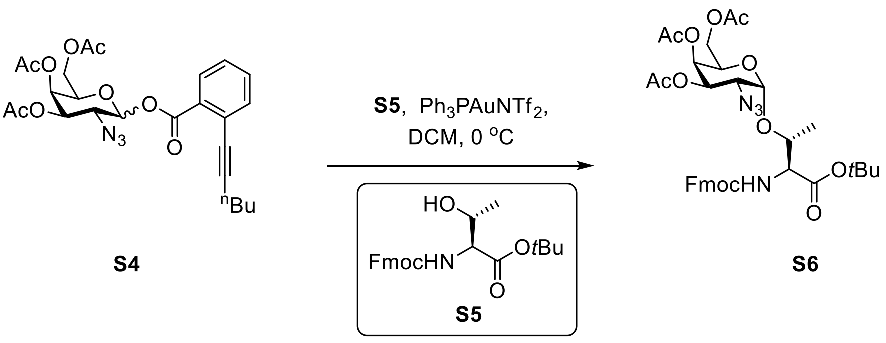

A solution of donor **S4** (10.9 g, 21.0 mmol, 1.0 equiv.) and acceptor **S5**[3] (12.5 g, 31.5 mmol, 1.5 equiv.) in dry DCM (300 mL) was stirred for 30 min at 0 °C with 4 Å molecular sieves under argon atmosphere.

To the solution $Ph_3PAuNTf_2$ (2.3 g, 3.2 mmol, 0.15 equiv.) was added. Then the solution was stirred at 0 °C for 1 h. Subsequently, the 4 Å molecular sieves was filtered off, and the filtrate was concentrated under reduced pressure. The residue was purified by flash chromatography to afford **S6** as a white solid (6.5 g, 9.7 mmol, 46%). ^{1}H NMR (400 MHz, Chloroform-*d*) δ 7.77 (d, *J* = 7.6 Hz, 2H), 7.63 (d, *J* = 7.7 Hz, 2H), 7.43 - 7.37 (m, 2H), 7.32 (dt, *J* = 11.2, 5.7 Hz, 2H), 5.67 (d, *J* = 9.5 Hz, 1H), 5.47 (d, *J* = 1.8 Hz, 1H), 5.34 (dd, *J* = 11.2, 3.3 Hz, 1H), 5.11 (d, *J* = 3.7 Hz, 1H), 4.46 - 4.25 (m, 6H), 4.10 (d, *J* = 6.5 Hz, 2H), 3.64 (dd, *J* = 11.2, 3.7 Hz, 1H), 2.15 (s, 3H), 2.08 (s, 3H), 2.05 (s, 3H), 1.50 (s, 9H), 1.39 - 1.32 (m, 3H).

Preparation of compound S7

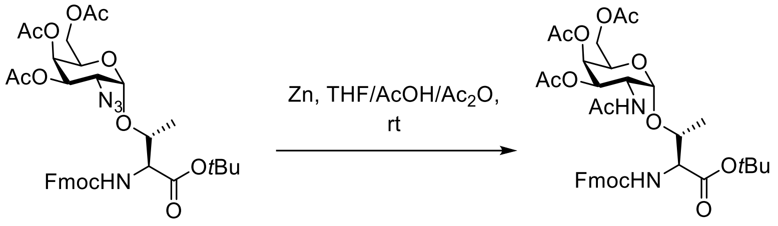

S6 **S7**

To the solution of **S6** (6.5 g, 9.7 mmol, 1.0 equiv.) in THF/AcOH/Ac_2O (3/2/1, v/v/v, 20 mL), activated zinc dust (7.6 g, 116.4 mmol, 12 equiv.) was added. Then the solution was stirred at room temperature for 3 h. Subsequently, the zinc dust was filtered off and the filtrate was concentrated. The residue solvent was diluted with DCM and washed with saturated $NaHCO_3$ solution. And then, the residue was concentrated and purified by flash chromatography to afford **S7** as a white solid (5.3 g, 7.2 mmol, 74%). ^{1}H NMR (400 MHz, Chloroform-*d*) δ 7.78 (d, *J* = 7.7 Hz, 2H), 7.64 (d, *J* = 7.4 Hz, 2H), 7.44 - 7.31 (m, 4H), 5.96 (d, *J* = 9.9 Hz, 1H), 5.52 (d, *J* = 9.6 Hz, 1H), 5.42 - 5.36 (m, 1H), 5.09 (dd, *J* = 11.5, 2.9 Hz, 1H), 4.92 - 4.86 (m, 1H), 4.62 (t, *J* = 10.8 Hz, 1H), 4.45 (q, *J* = 10.5, 9.3 Hz, 2H), 4.34 - 4.03 (m, 8H), 2.16 (s, 3H), 2.04 (s, 3H), 2.00 (s, 6H), 1.46 (s, 9H), 1.32 (d, *J* = 6.3 Hz, 3H).

Preparation of compound S8

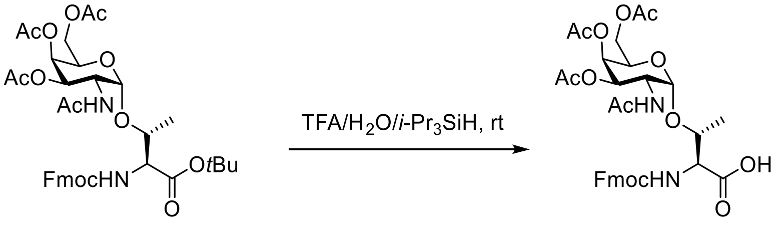

S7 **S8**

To the solution of Fmoc-Thr-(α-Ac_3GalNAc)-O*t*Bu **S7** (5.3 g, 7.2 mmol, 1.0 equiv.) in TFA (20 mL), triisopropylsilane (*i*-Pr_3SiH) (200 μL) and water (1 mL) were added. Then the solution was stirred at room temperature for 4 h. After concentrated to ~2 mL with a nitrogen flow, the residue solvent was azeotropically distilled with toluene. The residue was then purified by flash chromatography to afford compound **S8** as a white solid (4.0 g, 6.0 mmol, 83%). ^{1}H NMR (400 MHz, Chloroform-*d*) δ 7.75 (d, *J* = 11.5 Hz, 2H), 7.61 (s, 2H), 7.35 (d, *J* = 30.3 Hz, 4H), 6.29 (d, *J* = 10.2 Hz, 1H), 5.94 (d, *J* = 9.7 Hz, 1H), 5.40 - 5.32 (m, 1H), 5.14 (d, *J* = 11.9 Hz, 1H), 5.02 (d, *J* = 16.9 Hz, 2H), 4.40 (d, *J* = 22.4 Hz, 2H), 4.26 - 4.02 (m, 6H), 2.16 (s, 3H), 2.05 (s, 3H), 2.02 (s, 3H), 1.99 (s, 3H), 1.94 (s, 3H), 1.27 (d, *J* = 7.1 Hz, 3H).

3. Solid-phase peptide synthesis (SPPS)

Loading of hydrazine resin with Fmoc-Thr(*t*Bu)-OH and Fmoc-Ala-OH

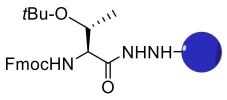

S9

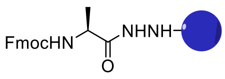

S10

2-Cl(Trt)-Cl resin was swollen in dry DCM for 30 min and treated with 10% hydrazine monohydrate in DMF for another 30 min. After that, the resin was washed with DMF, DCM. Then the resin was capped with MeOH by treating with a solution of DIPEA/ MeOH/ DCM (1/1/17, v/v/v) for 1.0 h. The resin was then washed with DCM, DMF. After that, Fmoc-protected amino acids Fmoc-Thr(*t*Bu)-OH and Fmoc-Ala-OH were loaded respectively by adding the solution of 1.0 equiv. each amino acid, HOAt and 2.0 equiv. DIPEA in DMF to the resin. After shaking for 1h, the resin was washed with DMF, DCM, DMF and the excessive hydrazine on the resin was capped with a solution of Ac_2O/DIPEA/DMF (1/2/2, v/v/v) for another 30 min. The resin was washed with DMF, DCM, MeOH, Et_2O and the residue solvent was removed *in vacuo* to give the hydrazide resin **S9** (0.42 mmol/g) and **S10** (0.33 mmol/g). The initial loading was measured by the UV-absorbance of the piperidine-fulvene adduct (λ = 301 nm, ε = 7800 $M^{-1}cm^{-1}$).

General procedures for SPPS

Fmoc-Deprotection: The hydrazine resin was deprotected by treating with 20% piperidine in DMF at 40 °C for 10 min twice. Then the resin was wash with DMF (3 times), DCM (3 times), DMF (3 times).

Coupling with normal amino acids: After Fmoc-deprotection, the hydrazine resin was treated with a solution of 8.0 equiv. Fmoc-protected amino acids [Fmoc-Pro-OH, Fmoc-Ala-OH, Fmoc-Gly-OH, Fmoc-Ser(*t*Bu)-OH, Fmoc-His(Trt)-OH, Fmoc-Val-OH, Fmoc-Asp(O*t*Bu)-OH, Fmoc-Arg(Pbf)-OH, Fmoc-Cys(S*t*Bu)-OH, Fmoc-His(Boc)-OH], HATU, 16.0 equiv. DIPEA in DMF (the concentration of amino acid: 0.25 M) at 40 °C for 30 min.

Coupling with Glycosylated amino acid S8/photo-cleavable moiety 4: After deprotection, the hydrazine resin was treated with a solution of compound **S8/4** (1.6 equiv.), HATU (1.6 equiv.), HOAt (1.6 equiv.) and DIPEA (3.2 equiv.) in DMF[5] (the concentration of amino acid: 0.2 M) at 40 °C for 30 min, and then added a solution of compound **S8/4** (0.4 equiv.), HATU (0.4 equiv.), HOAt (0.4 equiv.) and DIPEA (0.8 equiv.) in DMF (the concentration of amino acid: 0.2 M) to repeat the procedure.

Capping: The resin was washed by DMF (3 times) after coupling, and then treated with a solution of Ac_2O/lutidine/DMF (5/6/89, v/v/v) at 40 °C for 5 min to cap N-terminal residual amine. Then the resin was wash with DMF (3 times), DCM (3 times), DMF (3 times).

Solid phase synthesis of 5a on hydrazine resin S9 (0.05 mmol)

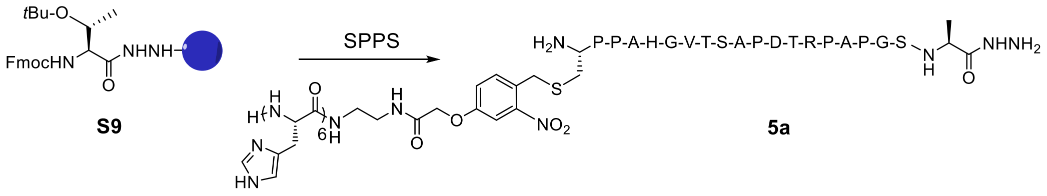

The peptide **5a** was synthesized according to the Fmoc-based SPPS outlined in the general procedures

above. After the last SPPS cycle, the resin was washed with DMF (3 × 5 mL), DCM (3 × 5 mL), MeOH (3 × 5 mL), Et_2O (3 × 5 mL) and dried. The resin bound peptides was cleaved with Reagent H[6] (2 mL) at room temperature for 2 h. The mixture was filtered and the resin was washed with Reagent H twice, and the filtrates were combined and concentrated to ~200 µL with a nitrogen flow. Then the residue was precipitated with ice-cold Et_2O. The mixture was filtered and the white filter cake was collected and dried *in vacuo* to give crude peptide **5a**. The crude product was purified by HPLC and lyophilized to afford a white powder (12.6 mg, 4.2 × 10^{-3} mmol, 8 % isolated yield).

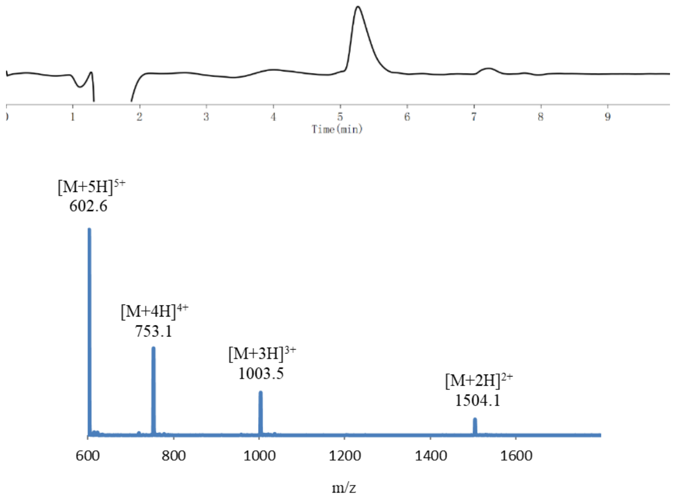

Figure S1. Analytical LCMS trace of the purified **5a**; A) Analytical HPLC: t_R = 5.3 min (2-30% solvent B in solvent A over 10 min, λ = 214 nm, C4 column); B) ESI-MS calculated for **5a** ($C_{127}H_{183}N_{48}O_{37}S$): $[M + 2H]^{2+}$ m/z = 1504.1, $[M + 3H]^{3+}$ m/z = 1003.1, $[M + 4H]^{4+}$ m/z = 752.1, $[M + 5H]^{5+}$ m/z = 602.2, found: 1504.1, 1003.5, 753.1, 602.6.

Solid phase synthesis of 5b on hydrazine resin S9 (0.05 mmol)

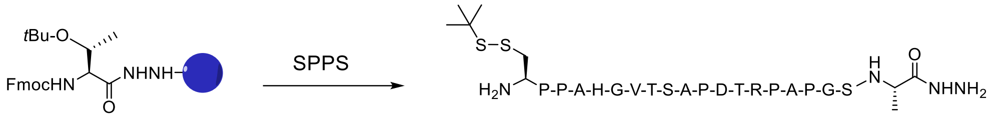

S9 **5b**

The peptide **5b** was synthesized according to the Fmoc-based SPPS outlined in the general procedures above. After the last SPPS cycle, the resin was washed with DMF (3 × 5 mL), DCM (3 × 5 mL), MeOH (3 × 5 mL), Et_2O (3 × 5 mL) and dried. The resin bound peptides was cleaved from the resin by treating with cocktail B (TFA/Phenol/H_2O/*i*-Pr_3SiH =88/5/5/2, v/v/v/v, 2mL) at room temperature for 2 h. The mixture was filtered and the resin was washed with cocktail B twice, and the filtrates were combined and concentrated to ~200 µL with a nitrogen flow. Then the residue was precipitated with ice-cold Et_2O. The mixture was filtered and the white filter cake was collected and dried *in vacuo* to give crude peptide **5b**. The crude product was purified by HPLC and lyophilized to afford a white powder (10.5 mg, 5.2 × 10^{-3} mmol, 10% isolated yield).

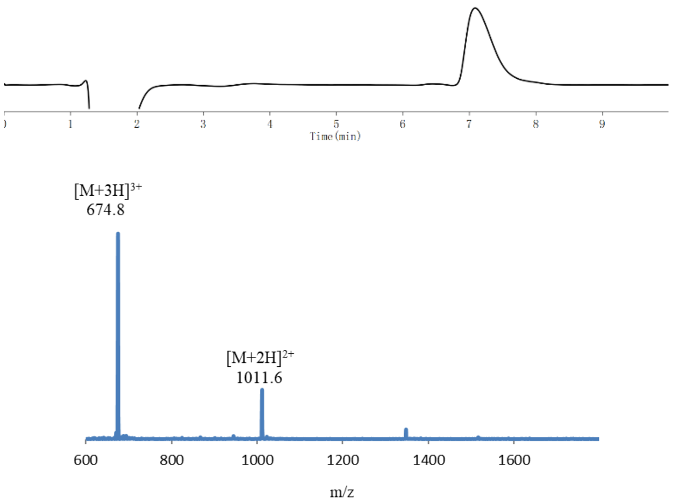

Figure S2. Analytical LCMS trace of the purified **5b**; A) Analytical HPLC: t_R = 7.1 min (2-30% solvent B in solvent A over 10 min, λ = 214 nm, C18 column); B) ESI-MS calculated for **5b** ($C_{84}H_{137}N_{27}O_{27}S_2$): $[M + 2H]^{2+}$ m/z = 1011.7, $[M + 3H]^{3+}$ m/z = 674.8, found: 1011.6, 674.8.

Solid phase synthesis of 8a on hydrazine resin S10 (0.132 mmol)

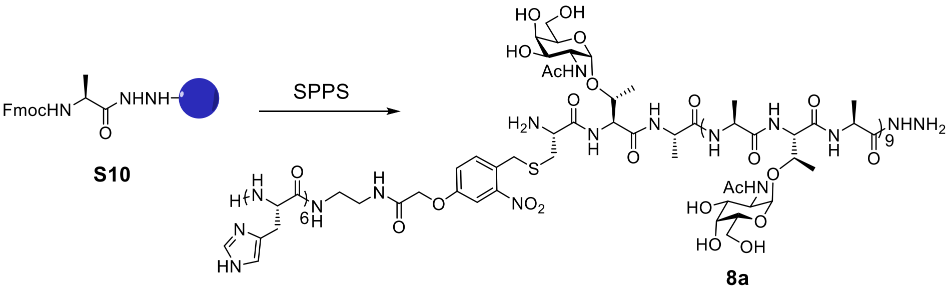

The peptide **8a** was synthesized according to the Fmoc-based SPPS outlined in the general procedures above. After the last SPPS cycle, the resin was washed with DMF (3 × 5 mL) and then treated with 10% hydrazine monohydrate in DMF to remove the acetyl protection on the saccharides. After 10 h then the resin was washed with DMF (3 × 5 mL), DCM (3 × 5 mL), MeOH (3 × 5 mL), Et_2O (3 × 5 mL) and dried. Subsequently, the resin bound peptide was cleaved with Reagent H[6] (3 mL) from resin at room temperature for 2 h. The mixture was filtered and the resin was washed with Reagent H twice, and the filtrates were combined and concentrated to ~200 μL with a nitrogen flow. Then the residue was precipitated with ice-cold Et_2O. The mixture was filtered and the white filter cake was collected and dried *in vacuo* to give crude peptide **8a**. The crude product was purified by HPLC and lyophilized to afford a white powder (279 mg, 0.06 mmol, 45% isolated yield).

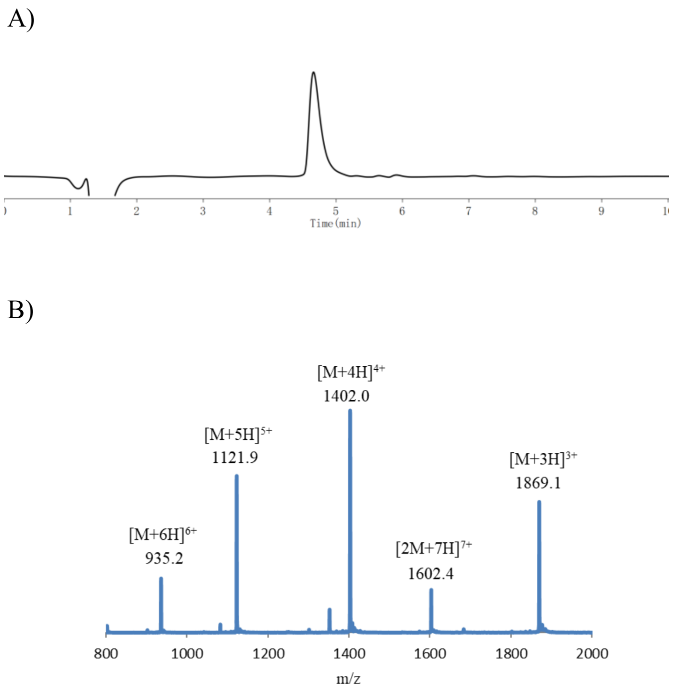

Figure S3. Analytical LCMS trace of the purified **8a**; A) Analytical HPLC: t_R = 4.7 min (5-30% solvent B in solvent A over 10 min, λ = 214 nm, C4 column); B) ESI-MS calculated for **8a** ($C_{227}H_{359}N_{63}O_{100}S$): [[M + 3H]$^{3+}$ m/z = 1868.6, [2M + 7H]$^{7+}$ m/z = 1601.8, [M + 4H]$^{4+}$ m/z = 1401.7, [M + 5H]$^{5+}$ m/z = 1121.6, [M + 6H]$^{6+}$ m/z = 934.8, found: 1869.1, 1602.4, 1402.0, 1121.9, 935.2.

Solid phase synthesis of 8b on hydrazine resin S10 (0.132 mmol)

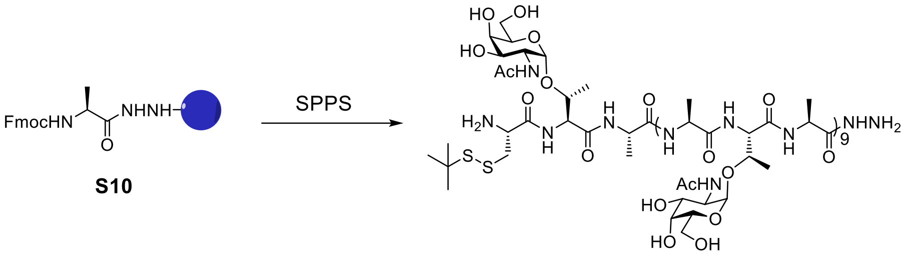

8b

The peptide **8b** was synthesized according to the Fmoc-based SPPS outlined in the general procedures above. After the last SPPS cycle, the resin was washed with DMF (3 × 5 mL) and then treated with 10% hydrazine monohydrate in DMF to remove the acetyl protection on the saccharides. After 10 h, the resin was washed with DMF (3 × 5 mL), DCM (3 × 5 mL), MeOH (3 × 5 mL), Et_2O (3 × 5 mL) and dried. Subsequently, the resin bound peptide was cleaved from the resin by treating with cocktail B (TFA/Phenol/H_2O/*i*-Pr_3SiH =8 8/5/5/2, v/v/v/v, 3 mL) at room temperature for 2 h. The mixture was filtered and the resin was washed with cocktail B twice, and the filtrates were combined and concentrated to ~200 μL with a nitrogen flow. Then the residue was precipitated with ice-cold Et_2O. The mixture was filtered and the white filter cake was collected and dried *in vacuo* to give crude peptide **8b**. The crude product was purified by HPLC and lyophilized to afford a white powder (279 mg, 0.06 mmol, 45% isolated yield).

A)

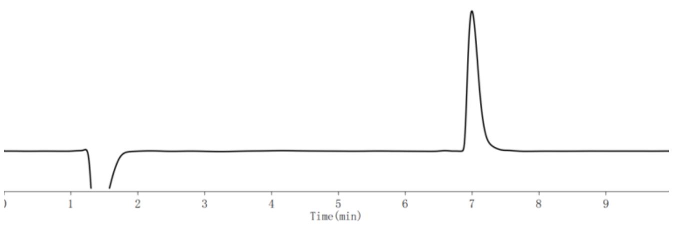

B)

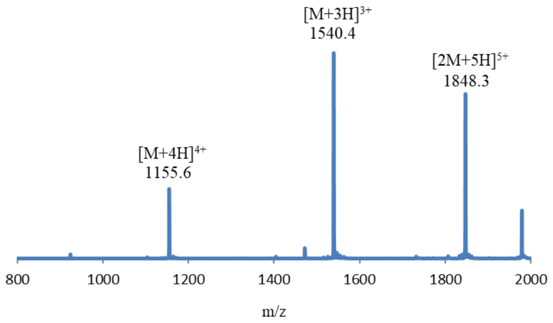

Figure S4. Analytical LCMS trace of the purified **8b**; A) Analytical HPLC: t_R = 7.1 min (5-30% solvent B in solvent A over 10 min, λ = 214 nm, C4 column); B) ESI-MS calculated for **8b** ($C_{184}H_{312}N_{42}O_{90}S_2$): $[2M + 5H]^{5+}$ m/z = 1849.0, $[M + 3H]^{3+}$ m/z = 1540.0, $[M + 4H]^{4+}$ m/z = 1155.2, found: 1848.3, 1540.4, 1155.6.

Solid phase synthesis of 13c on hydrazine resin S10 (0.012 mmol)

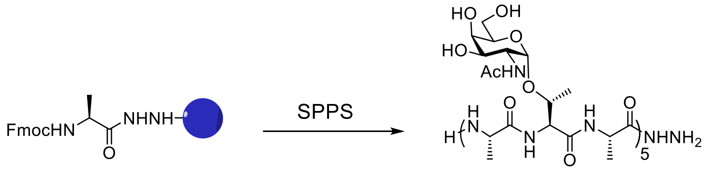

S10 **13c**

The AFGP mimic **13c** was synthesized on a preloaded resin **S10** (0.012 mmol) according to Fmoc-strategy SPPS protocol (See general procedures for SPPS). After the last SPPS cycle, the resin was washed with DMF (3 × 5 mL) and then treated with 10% hydrazine monohydrate in DMF to remove the acetyl protection on the saccharides. After 10 h, the resin was washed with DMF (3 × 3 mL), DCM (3 × 3 mL), MeOH (3 × 3 mL), Et_2O (3 × 3 mL) and dried. The resin was treated with Cocktail B (TFA/Phenol/H_2O/*i*-Pr_3SiH =8 8/5/5/2, v/v/v/v, 3 mL) at room temperature for 2 h. The mixture was filtered and the resin was washed with cocktail B twice, and the filtrates were combined and concentrated to ~100 µL with a nitrogen flow, and the residue was precipitated with ice-cold Et_2O. The white solid was filtered and dried *in vacuo* to give crude peptide **13c**. The crude peptide was purified by HPLC and lyophilized to afford a white powder (12 mg, 0.005 mmol, 42% isolated yield).

A)

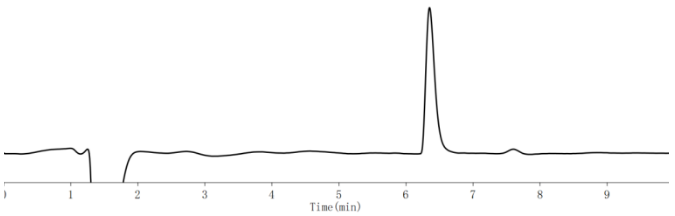

B)

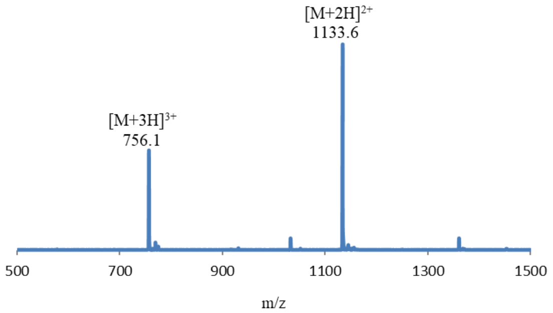

Figure S5. Analytical LCMS trace of the purified **13c**; A) Analytical HPLC: t_R = 6.4 min (2-20% Solvent B in solvent A over 10 min, λ= 214 nm, C18 column); B) ESI-MS calculated for **13c** ($C_{90}H_{154}N_{22}O_{45}$): $[M + 2H]^{2+}$ m/z = 1133.2, $[M + 3H]^{3+}$ m/z = 755.8, found: 1133.6, 756.1.

4. The assembly of peptide segments

4.1 General procedures for peptide assembly

Activation of peptide hydrazide to thioester and the subsequent NCL: To a solution (6 M Gnd·HCl, 0.2 M phosphate, 0.2 M MPAA, pH 3) of peptide hydrazide **5a-10a** (1.8 mM, 1.0 equiv.), a solution of acetylacetonate (150 mM, 2.0 equiv.) was added. After vigorously stirring at room temperature for 5-10 h, the reaction was monitored by LC-MS. After completion, the solution was adjusted to pH 6.5 and mixed with the solution (6 M Gnd·HCl, 0.2 M phosphate, 20 mM TCEP, pH 6.5, 1/3 volume of thioester solution) of another peptide segment **5b-10b** with a free N-terminal cysteine (7 mM, 1.2-1.5 equiv.). The mixture was degassed by sparging with argon, and then stirred at 37 °C for 1 h.

Purification of NCL product with Ni-NTA resin: Ni-NTA resin (50% suspension, 3.3 × 10^{-4} mmol/mL, 1.0 equiv.) was washed and equilibrated with washing buffer (6 M Gnd·HCl, 0.2 M phosphate, pH 7) for 5 min. Then the NCL reaction solution was added to the beads. A gentle shaking was performed until no ligation product left in the solution monitored by LC-MS. The Ni-NTA resin was washed three times with washing buffer (6 M Gnd·HCl, 0.2 M phosphate, pH 7) and distilled water, respectively. Subsequently, the peptide hydrazide containing His_6 tag was eluted in an acidic solution (6 M Gnd·HCl, 0.2 M phosphate, pH 3) which was directly used in the next thioesterification cycle or PPH removal.

Removal of PPH: To the elution of Ni-NTA resin from last procedure, the semicarbazide hydrochloride

(50 mM) and DTT (50 mM) were added. After degassed with a steady flow of argon for 10 min, the solution was irradiated with UV light (365 nm, 20 W). The reaction was completed in 15 min, and then the product was purified with HPLC and lyophilized to yield a white solid.

4.2 Synthetic Details and Analytical data

4.2.1 Activation of peptide hydrazide 5a to thioester 5_{SR}:

To a solution (6 M Gnd·HCl, 0.2 M phosphate, 0.2 M MPAA, pH 3, 1.6 mL) of peptide hydrazide **5a** (10 mg, 2.9×10^{-3} mmol, 1.0 equiv.), a solution of acetylacetonate (150 mM, 2.0 equiv., 39 µL) was added. The resulting mixture was vigorously stirred at room temperature for 5 h to give $\mathbf{5_{SR}}$. After completion monitored by LC-MS, the yield was calculated by peak-integration of the HPLC profile (λ = 214 nm) (2.9×10^{-3} mmol, > 99% HPLC yield).

A)

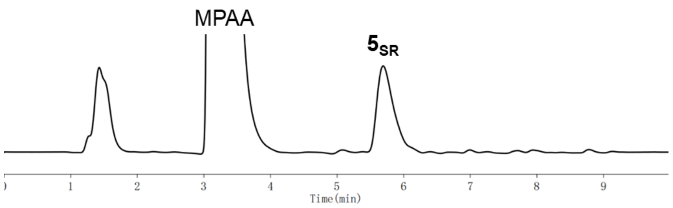

B)

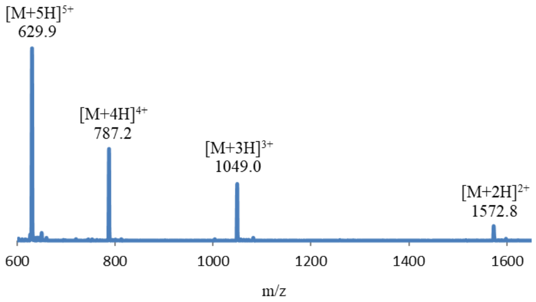

Figure S6. Analytical LCMS trace of the crude $\mathbf{5_{SR}}$; A) Analytical HPLC: t_R = 5.7 min (2-30% solvent B in solvent A over 10 min, λ = 214 nm, C4 column); B) ESI-MS calculated for $\mathbf{5_{SR}}$ ($C_{135}H_{187}N_{46}O_{39}S_2$): $[M + 2H]^{2+}$ m/z = 1572.2, $[M + 3H]^{3+}$ m/z = 1048.5, $[M + 4H]^{4+}$ m/z = 786.6, $[M + 5H]^{5+}$ m/z = 629.5, found: 1574.8, 1049.0, 787.2, 629.9.

4.2.2 Synthesis of 6a *via* ligation of 5_{SR} and 5b and the subsequent Ni-NTA purification

The solution (from **4.2.1**) containing $\mathbf{5_{SR}}$ (2.9×10^{-3} mmol, 1.0 equiv.) was adjusted to pH 6.5 and mixed with the solution (6 M Gnd·HCl, 0.2 M phosphate, 20 mM TCEP, pH 6.5, 500 µL) of peptide **5b** (3.5×10^{-3} mmol, 7.0 mM, 1.2 equiv., 7.1 mg). The mixture was degassed by sparging with argon and stirred at 37 °C for 1 h. Subsequently, the crude product **6a** was purified with Ni-NTA resin as stated in general procedures for peptide assembly. The acidic elution was then directly used in the next thioesterification cycle or PPH removal. The yield was calculated by peak-integration of the HPLC profile (λ = 214 nm) (2.6×10^{-3} mmol, 89% HPLC yield).

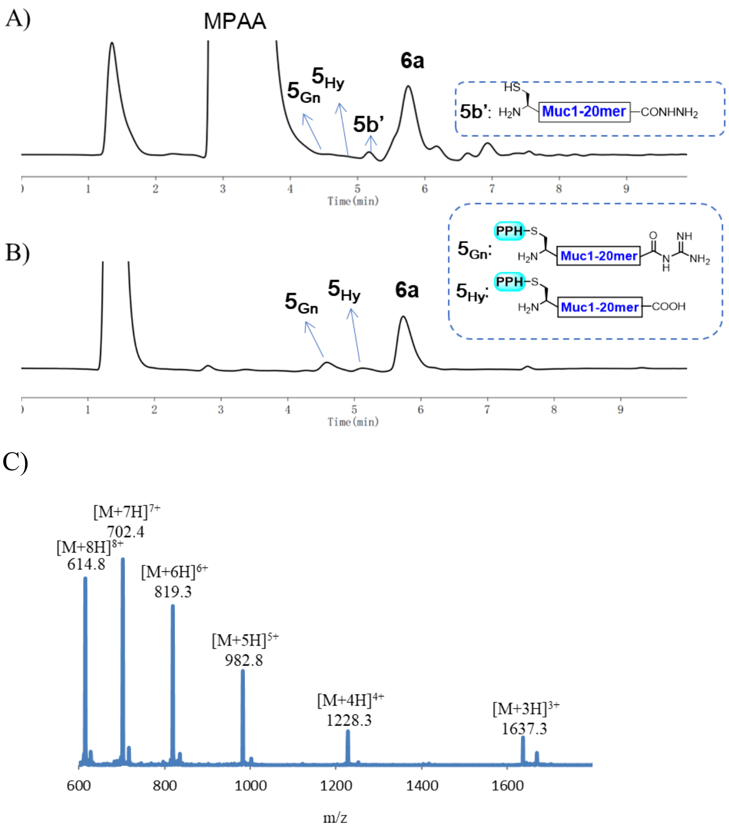

Figure S7. Analytical LCMS trace of **6a**; A) and B): Analytical HPLC of crude and Ni-NTA resin purified **6a**, t_R = 5.8 min (2-30% solvent B in solvent A over 10 min, λ = 214 nm, C4 column); C) ESI-MS calculated for **6a** ($C_{207}H_{308}N_{73}O_{64}S_2$): $[M + 3H]^{3+}$ m/z = 1636.8, $[M + 4H]^{4+}$ m/z = 1227.8, $[M + 5H]^{5+}$ m/z = 982.5, $[M + 6H]^{6+}$ m/z = 818.9, $[M + 7H]^{7+}$ m/z = 702.0, $[M + 8H]^{8+}$ m/z = 614.4, found: 1637.3, 1228.3, 982.2, 819.3, 702.4, 614.8.

4.2.3 PPH removal of 6a to yield 40mer 6b

To the acidic solution (5 mL, from **4.2.2**) with eluted **6a** (1.9×10^{-3} mmol) from Ni-NTA resin, semicarbazide hydrochloride (50 mM, 0.25 mmol, 28 mg) and DTT (50 mM, 0.25 mmol, 39 mg) were added. After degassed with a steady flow of argon for 10 min, the solution was irradiated with UV light (365 nm, 20 W). The reaction was completed in 15 min, and then the product was purified with HPLC and lyophilized to yield a white solid **6b** (4.0 mg, 1.0×10^{-3} mmol, 52% isolated yield from **5a**).

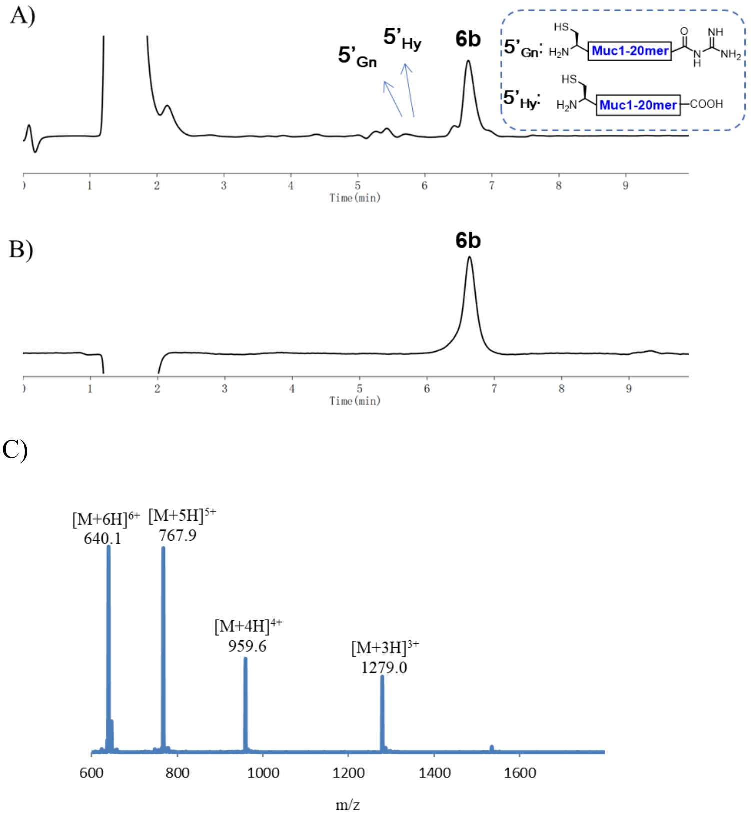

Figure S8. Analytical LCMS trace of **6b**; A) and B): Analytical HPLC of crude and HPLC purified **6b**, t_R = 6.6 min (2-30% solvent B in solvent A over 10 min, λ = 214 nm, C4 column); C) ESI-MS calculated for **6b** ($C_{207}H_{308}N_{73}O_{64}S_2$): $[M + 3H]^{3+}$ m/z = 1279.1, $[M + 4H]^{4+}$ m/z = 959.6, $[M + 5H]^{5+}$ m/z = 767.9, $[M + 6H]^{6+}$ m/z = 640.1, found: 1279.1, 959.6, 767.9, 640.1.

4.2.4 Activation of peptide hydrazide 6a to thioester 6_{SR}:

The solution (from **4.2.2**) with of eluted **6a** (7.0×10^{-4} mmol) from the Ni-NTA resin was concentrated to 380 µL by ultrafiltration (Millipore MWCO 3000), MPAA (0.2 M, 12 mg, 0.076 mmol) and a solution of acetylacetonate (150 mM, 2.0 equiv., 9 µL) were then added. The mixture was vigorously stirred after adjustment of pH to 3.0, and the reaction was monitored by LC-MS. The conversion was completed in 5 h to give $\mathbf{6_{SR}}$. The yield was calculated by peak-integration of the HPLC profile (λ = 214 nm) (7.0×10^{-4} mmol, > 99% HPLC yield).

A)

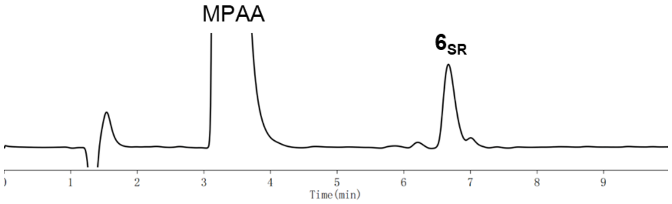

B)

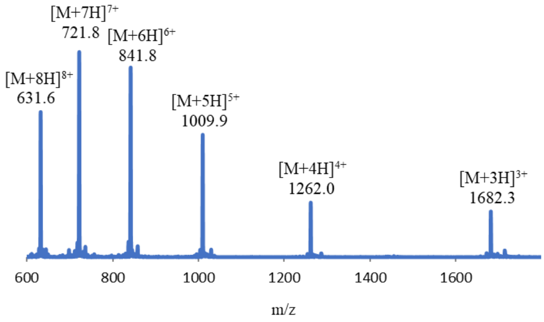

Figure S9. Analytical LCMS trace of the crude **6**$_{\mathbf{SR}}$; A) Analytical HPLC: t_R = 6.6 min (2-30% solvent B in solvent A over 10 min, λ = 214 nm, C4 column); B) ESI-MS calculated for **6**$_{\mathbf{SR}}$ ($C_{215}H_{312}N_{71}O_{66}S_3$): $[M + 3H]^{3+}$ m/z = 1682.2, $[M + 4H]^{4+}$ m/z = 1261.9, $[M + 5H]^{5+}$ m/z = 1009.7, $[M + 6H]^{6+}$ m/z = 841.6, $[M + 7H]^{7+}$ m/z = 721.5, $[M + 8H]^{8+}$ m/z = 631.4, found: 1682.3, 1262.0, 1009.9, 841.8, 721.8, 631.6.

4.2.5 Synthesis of 7a *via* ligation of 6$_{\mathbf{SR}}$ **and 6b and the subsequent Ni-NTA purification**

The solution (from **4.2.4**) containing **6**$_{\mathbf{SR}}$ (7.0×10^{-4} mmol, 1.0 equiv.) was adjusted to pH 6.5 and mixed with the solution (6 M Gnd·HCl, 0.2 M phosphate, 20 mM TCEP, pH 6.5, 6 mL) of peptide **6b** (1.0×10^{-3} mmol, 7.0 mM, 1.5 equiv., 4.0 mg). The mixture was degassed by sparging with argon and stirred at 37 °C for 1 h. Subsequently, the crude product **7a** was purified with Ni-NTA resin as stated in general procedures for peptide assembly. The acidic elution was then directly used in the next thioesterification cycle or PPH removal. The yield was calculated by peak-integration of the HPLC profile (λ = 214 nm) (5.6×10^{-4} mmol, 80% HPLC yield).

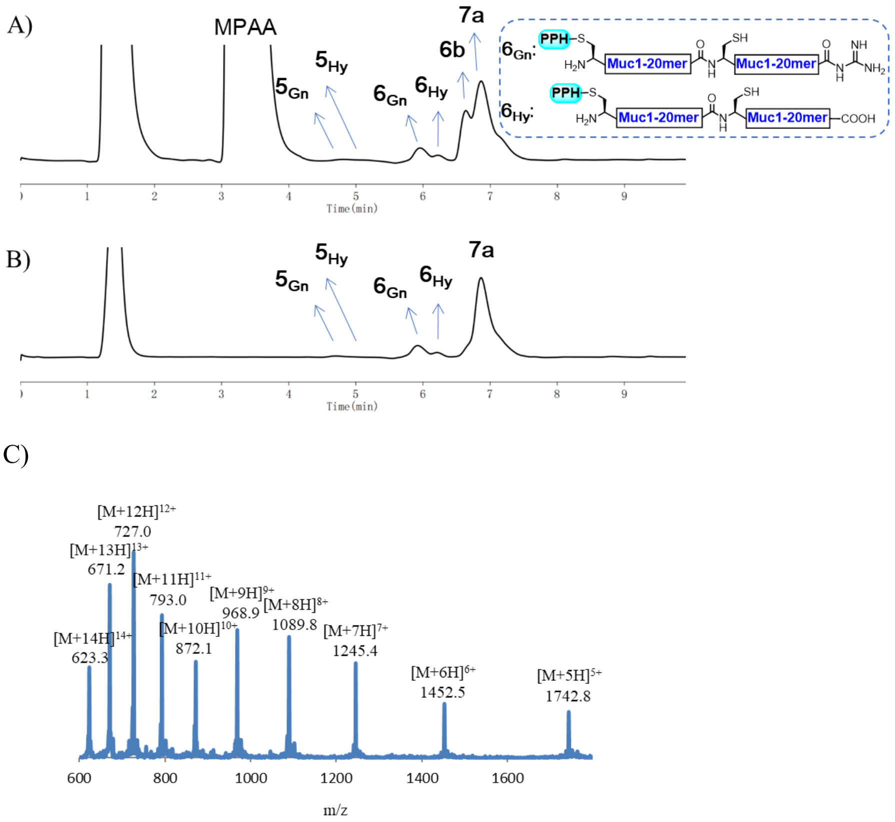

Figure S10. Analytical LCMS trace of **7a**; A) and B): Analytical HPLC of crude and Ni-NTA resin purified **7a**, t_R = 6.9 min (2-30% solvent B in solvent A over 10 min, λ = 214 nm, C4 column); C) ESI-MS calculated for **7a** ($C_{367}H_{558}N_{123}O_{118}S_4$): $[M + 5H]^{5+}$ m/z = 1742.9, $[M + 6H]^{6+}$ m/z = 1452.6, $[M + 7H]^{7+}$ m/z = 1245.2, $[M + 8H]^{8+}$ m/z = 1089.7, $[M + 9H]^{9+}$ m/z = 968.7, $[M + 10H]^{10+}$ m/z = 872.0, $[M + 11H]^{11+}$ m/z = 792.8, $[M + 12H]^{12+}$ m/z = 726.8, $[M + 13H]^{13+}$ m/z = 671.0, $[M + 14H]^{14+}$ m/z = 623.1, found: 1742.8, 1452.5, 1245.4, 1089.8, 968.9, 872.1, 793.0, 727.0, 671.2, 623.3.

4.2.6 PPH removal of 7a to yield 80mer 7b

To the acidic solution (1 mL, from **4.2.5**) with eluted **7a** (5.6×10^{-4} mmol) from Ni-NTA resin, semicarbazide hydrochloride (50 mM, 0.25 mmol, 5.6 mg) and DTT (50 mM, 0.25 mmol, 7.8 mg) were added. After degassed with a steady flow of argon for 10 min, the solution was irradiated with UV light (365 nm, 20 W). The reaction was completed in 15 min, and then the product was purified with HPLC and lyophilized to yield a white solid **7b** (2.2 mg, 2.5×10^{-3} mmol, 30% isolated yield from **5a**).

A)

B)

C)

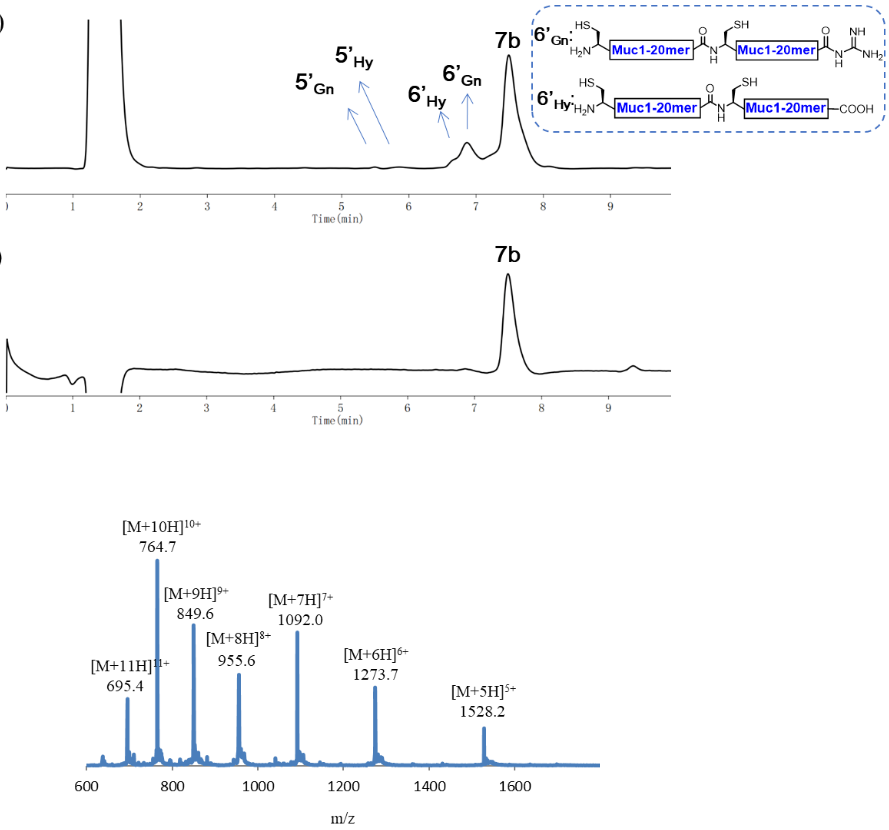

Figure S11. Analytical LCMS trace of **6b**; A) and B): Analytical HPLC of crude and HPLC purified **6b**, t_R = 7.5min (2-30% solvent B in solvent A over 10 min, λ = 214 nm, C4 column); C) ESI-MS calculated for **6b** ($C_{320}H_{504}N_{102}O_{108}S_4$): $[M + 5H]^{5+}$ m/z = 1528.3, $[M + 6H]^{6+}$ m/z = 1273.7, $[M + 7H]^{7+}$ m/z = 1091.9, $[M + 8H]^{8+}$ m/z = 955.6, $[M + 9H]^{9+}$ m/z = 849.5, $[M + 10H]^{10+}$ m/z = 764.6, $[M + 11H]^{11+}$ m/z = 695.2, found: 1528.2, 1273.7, 1092.0, 955.6, 849.6, 764.7, 695.4.

4.2.7 Activation of peptide hydrazide 8a to thioester 8_{SR}:

To a solution (6 M Gnd·HCl, 0.2 M phosphate, 0.2 M MPAA, pH 3, 19 mL) of peptide hydrazide **8a** (196 mg, 0.035 mmol, 1.0 equiv.), a solution of acetylacetonate (150 mM, 2.0 equiv., 470 μL) was added. The resulting mixture was vigorously stirred at room temperature for 10 h. After completion monitored by LC-MS, the yield was calculated by peak-integration of the HPLC profile (λ = 214 nm) (0.035 mmol, > 99% HPLC yield).

A)

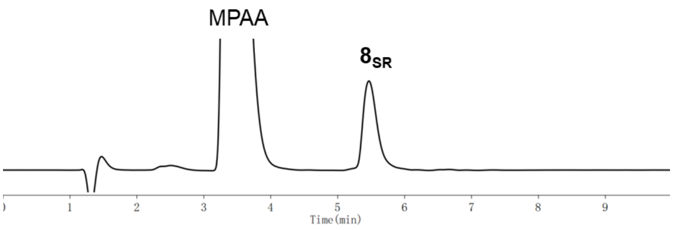

B)

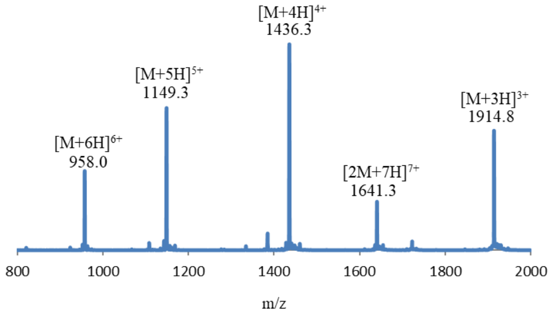

Figure S12. Analytical LCMS trace of the crude **8_{SR}**; A) Analytical HPLC: t_R = 5.5 min (5-30% solvent B in solvent A over 10 min, λ = 214 nm, C4 column); B) ESI-MS calculated for **8_{SR}** ($C_{235}H_{363}N_{61}O_{102}S_2$): $[M + 3H]^{3+}$ m/z = 1914.0, $[2M + 7H]^{7+}$ m/z = 1640.7, $[M + 4H]^{4+}$ m/z = 1435.7, $[M + 5H]^{5+}$ m/z = 1148.8, $[M + 6H]^{6+}$ m/z = 957.5, found: 1914.8, 1641.3, 1436.3, 1149.3, 958.0.

4.2.8 Synthesis of 9a *via* ligation of 8_{SR} and 8b and the subsequent Ni-NTA purification

The solution (from **4.2.7**) containing **8_{SR}** (0.035 mmol, 1.0 equiv.) was adjusted to pH 6.5 and mixed with the solution (6 M Gnd·HCl, 0.2 M phosphate, 20 mM TCEP, pH 6.5, 6 mL) of peptide **8b** (0.042 mmol, 7.0 mM, 1.2 equiv., 194 mg). The mixture was degassed by sparging with argon and stirred at 37 °C for 1 h. Subsequently, the crude product **9a** was purified with Ni-NTA resin as stated in general procedures for peptide assembly. The acidic elution was then directly used in the next thioesterification cycle or PPH removal. The yield was calculated by peak-integration of the HPLC profile (λ = 214 nm) (0.035 mmol, > 99% HPLC yield).

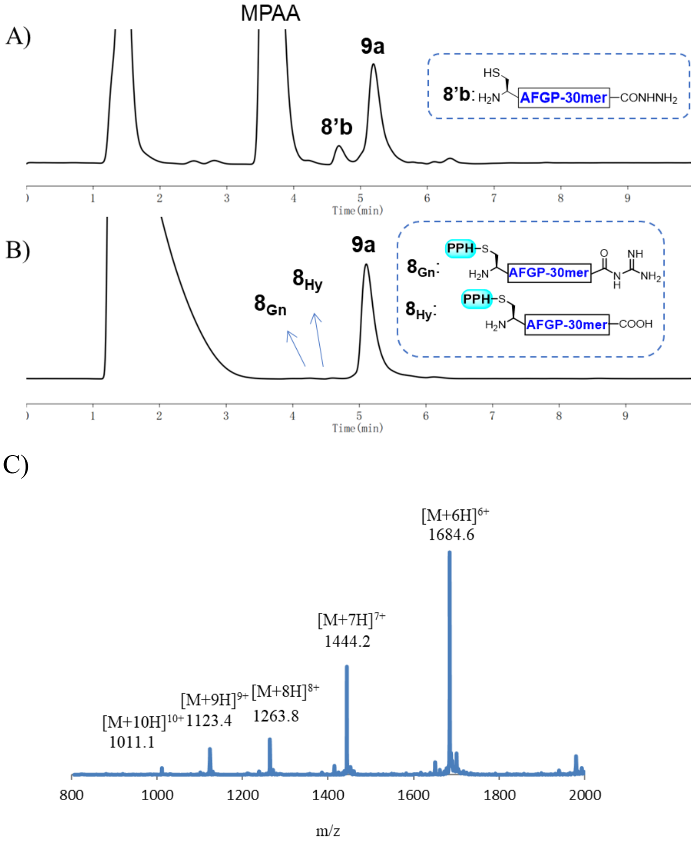

Figure S13. Analytical LCMS trace of **9a**; A) and B): Analytical HPLC of crude and Ni-NTA resin purified **9a**, t_R = 5.2 min (5-30% solvent B in solvent A over 10 min, λ = 214 nm, C4 column); C) ESI-MS calculated for **9a** ($C_{407}H_{659}N_{103}O_{190}S_2$): $[M + 6H]^{6+}$ m/z = 1684.2, $[M + 7H]^{7+}$m/z = 1443.8, $[M + 8H]^{8+}$ m/z = 1263.4, $[M + 9H]^{9+}$ m/z = 1123.4, $[M + 10H]^{10+}$ m/z = 1010.9, found: 1684.6, 1444.2, 1263.8, 1123.4, 1011.1.

4.2.9 PPH removal of 9a to yield 60mer 9b

To the solution (100 mL, from **4.2.8**) with eluted **9a** (0.028 mmol) from Ni-NTA resin, semicarbazide hydrochloride (50 mM, 5 mmol, 558 mg) and DTT (50 mM, 5 mmol, 771 mg) were added. After degassed with a steady flow of argon for 10 min, the solution was irradiated with UV light (365 nm, 20 W). The reaction was completed in 15 min, and then the product was purified with HPLC and lyophilized to yield a white solid **9b** (86 mg, 9.5 × 10^{-3} mmol, 34% isolated yield from **8a**).

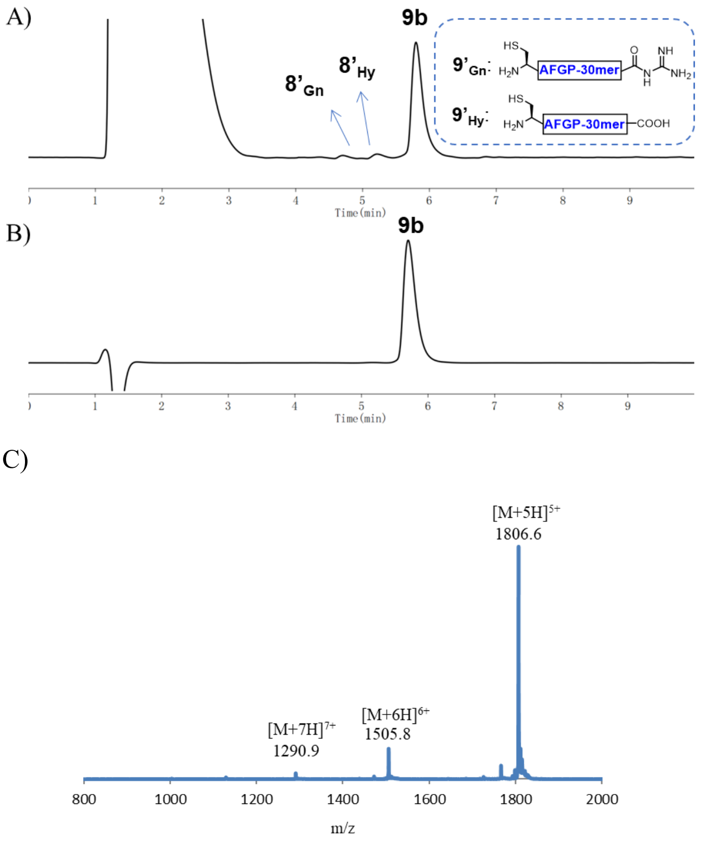

Figure S14. Analytical LCMS trace of **9b**; A) and B): Analytical HPLC of crude and HPLC purified **9b**, t_R = 5.8 min (5-30% solvent B in solvent A over 10 min, λ = 214 nm, C4 column); C) ESI-MS calculated for **9b** ($C_{360}H_{604}N_{82}O_{180}S_2$): $[M + 5H]^{5+}$ m/z = 1806.1, $[M + 6H]^{6+}$ m/z = 1505.2, $[M + 7H]^{7+}$ m/z = 1290.3, found: 1806.6, 1505.8, 1290.9.

4.2.10 Activation of peptide hydrazide 9a to thioester 9$_{SR}$:

The solution (from **4.2.8**) with of eluted **9a** (6.6 × 10^{-3} mmol) from the Ni-NTA resin was concentrated to 3.0 mL by ultrafiltration (Millipore MWCO 3000). After that, MPAA (0.2 M, 101 mg, 0.6 mmol) and a solution of acetylacetonate (150 mM, 2.0 equiv., 88 μL) were then added. The mixture was vigorously stirred after adjustment of pH to 3.0, and the reaction was monitored by LC-MS. The conversion was completed in 10 h. The yield was calculated by peak-integration of the HPLC profile (λ = 214 nm) (6.6 × 10^{-3} mmol, > 99% HPLC yield).

A)

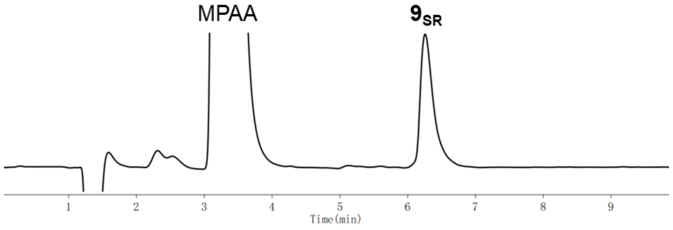

B)

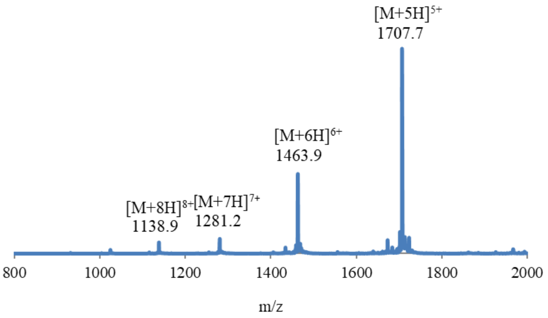

Figure S15. Analytical LCMS trace of the crude **9$_{SR}$**; A) Analytical HPLC: t_R = 6.3 min (5-30% solvent B in solvent A over 10 min, λ = 214 nm, C4 column); B) ESI-MS calculated for **9$_{SR}$** ($C_{415}H_{663}N_{101}O_{192}S_3$): $[M + 5H]^{5+}$ m/z = 1706.9, $[M + 6H]^{6+}$ m/z = 1463.2, $[M + 7H]^{7+}$ m/z = 1280.4, $[M + 8H]^{8+}$ m/z = 1138.3, found: 1707.7, 1463.9, 1281.2, 1138.9.

4.2.11 Synthesis of 8a *via* ligation of 9$_{SR}$ and 9b and the subsequent Ni-NTA purification

The solution (from **4.2.10**) containing **9$_{SR}$** (6.6×10^{-3} mmol, 1.0 equiv.) was adjusted to pH 7 and mixed with the solution (6 M Gnd·HCl, 0.2 M phosphate, 20 mM TCEP, pH 6.5, 1 mL) of peptide **9b** (7.9×10^{-3} mmol 7.9 mM, 1.2 equiv., 71 mg). The mixture was degassed by sparging with argon and stirred at 37 °C for 1 h. Subsequently, the crude product **10a** was purified with Ni-NTA resin as stated in general procedures for peptide assembly. The acidic elution was then directly used in the next thioesterification cycle or PPH removal. The yield was calculated by peak-integration of the HPLC profile (λ = 214 nm) (4.0×10^{-3} mmol, 61% HPLC yield).

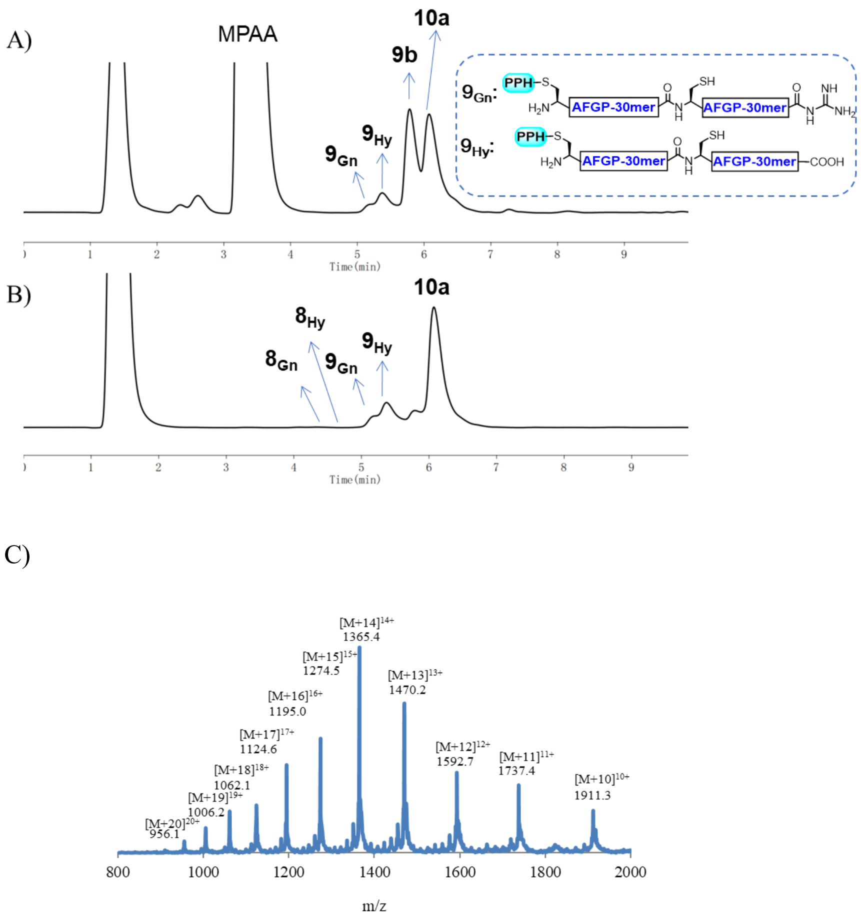

Figure S16. Analytical LCMS trace of **10a**; A) and B): Analytical HPLC of crude and Ni-NTA resin purified **10a**, t_R = 6.2 min (5-30% solvent B in solvent A over 10 min, λ = 214 nm, C4 column); C) ESI-MS calculated for **10a** ($C_{767}H_{1259}N_{183}O_{370}S_4$): $[M + 10H]^{10+}$ m/z = 1910.3, $[M + 11H]^{11+}$ m/z = 1736.7, $[M + 12H]^{12+}$ m/z = 1592.1, $[M + 13H]^{13+}$ m/z = 1470.0, $[M + 14H]^{14+}$ m/z = 1364.8, $[M + 15H]^{15+}$ m/z = 1274.5, $[M + 16H]^{16+}$ m/z = 1194.3, $[M + 17H]^{17+}$ m/z = 1124.1, $[M + 18H]^{18+}$ m/z = 1061.7, $[M + 19H]^{19+}$ m/z = 1005.9, $[M + 20H]^{20+}$ m/z = 955.6, found: 1911.3, 1737.4, 1592.7, 1470.2, 1365.4, 1274.5, 1195.0, 1124.6, 1062.1, 1006.2, 956.1.

4.2.12 Removal of PPH to yield 120mer peptide 10b

To the solution (17 mL, from **4.2.11**) of eluted **10a** (2.65 × 10^{-3} mmol) from Ni-NTA resin, the semicarbazide hydrochloride (50 mM, 0.85 mmol, 94 mg) and DTT (50 mM, 0.85 mmol, 131 mg) were added. After degassed with a steady flow of argon for 10 min, the solution was irradiated with UV light (365 nm, 20 W). The reaction was completed in 15 min, and then the product was purified with HPLC and lyophilized to yield a white solid **10b** (15 mg, 8.4 × 10^{-4} mmol, 19% isolated yield from **8a**).

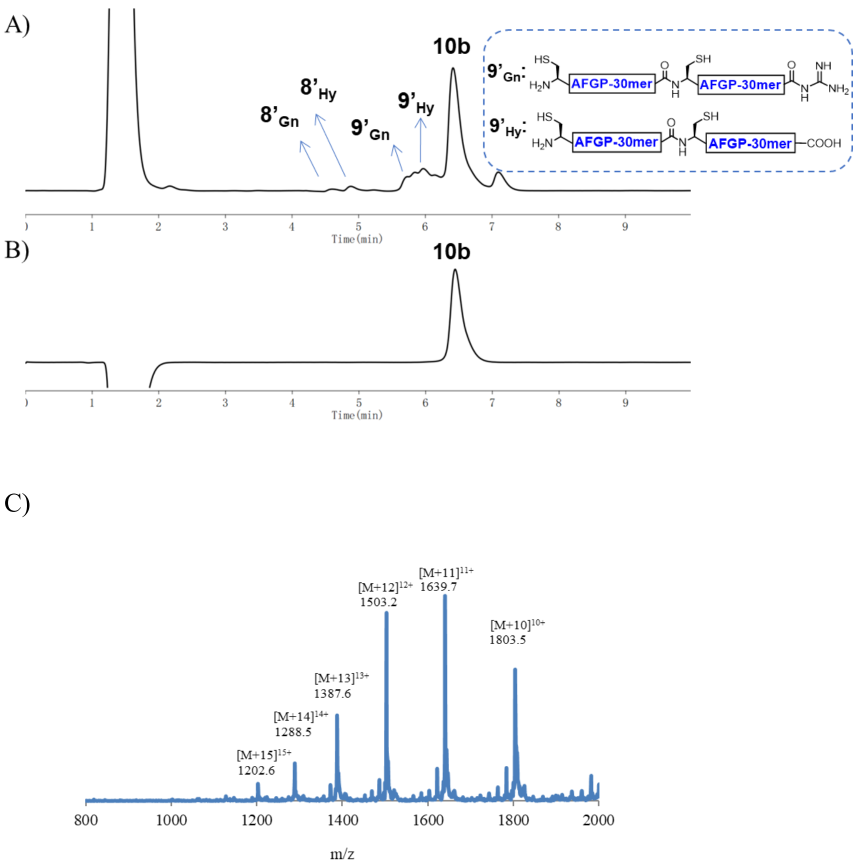

Figure S17. Analytical LCMS trace of **10b**; A) and B): Analytical HPLC of crude and HPLC purified **10b**, t_R = 6.5 min (5-30% solvent B in solvent A over 10 min, λ = 214 nm, C4 column); C) ESI-MS calculated for **10b** ($C_{720}H_{1204}N_{162}O_{360}S_4$): $[M + 10H]^{10+}$ m/z = 1802.9, $[M + 11H]^{11+}$ m/z = 1639.1, $[M + 12H]^{12+}$ m/z = 1502.5, $[M + 13H]^{13+}$ m/z = 1387.0, $[M + 14H]^{14+}$ m/z = 1288.0, $[M + 15H]^{15+}$ m/z = 1202.2, found: 1803.5, 1639.7, 1503.2, 1387.6, 1288.5, 1202.6.

4.2.13 Activation of peptide hydrazide 10a to thioester 10_{SR}:

The solution (from **4.2.11**) of eluted **10a** (1.14×10^{-3} mmol, 1.0 equiv.) from the Ni-NTA resin was concentrated to 200 μL by ultrafiltration (Millipore MWCO 10000). And then, MPAA (0.2 M, 6.7 mg, 0.2 mmol) and a solution of acetylacetonate (150 mM, 2.0 equiv., 15.2 μL) were added. After vigorously stirred at room temperature for 10 h after adjustment of pH to 3.0, the reaction was monitored by LC-MS and the conversion was completed. The yield was calculated by peak-integration of the HPLC profile (λ = 214 nm) (9.2×10^{-4} mmol, 80% HPLC yield).

A)

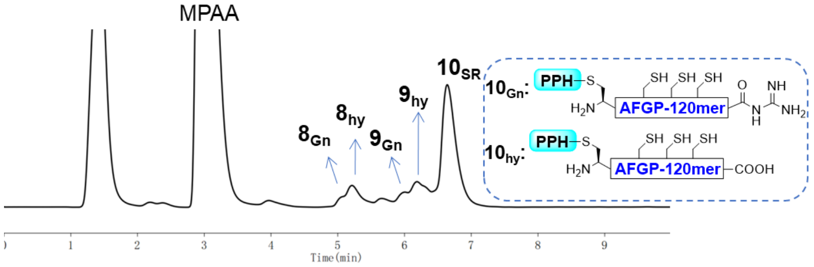

B)

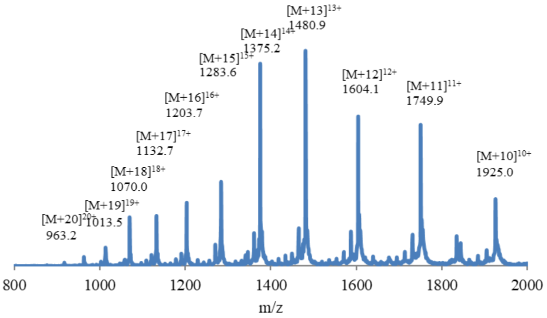

Figure S18. Analytical LCMS trace of the crude **10_{SR}**; A) Analytical HPLC: t_R = 6.8 min (5-30% solvent B in solvent A over 10 min, λ = 214 nm, C4 column); B) ESI-MS calculated for **10_{SR}** ($C_{775}H_{1263}N_{181}O_{372}S_5$): $[M + 10H]^{10+}$ m/z = 1923.9., $[M + 11H]^{11+}$ m/z = 1749.1, $[M + 12H]^{12+}$ m/z = 1603.4, $[M + 13H]^{13+}$ m/z = 1480.1, $[M + 14H]^{14+}$ m/z = 1374.5, $[M + 15H]^{15+}$ m/z = 1282.9, $[M + 16H]^{16+}$ m/z = 1202.8, $[M + 17H]^{17+}$ m/z = 1132.1, $[M + 18H]^{18+}$ m/z = 1069.3, $[M + 19H]^{19+}$ m/z = 1013.1, $[M + 20H]^{20+}$ m/z = 962.4, found: 1925.0, 1749.9, 1604.1, 1480.9, 1375.2, 1283.6, 1203.7, 1132.7, 1070.0, 1013.5, 963.2.

4.2.14 Synthesis of 11a *via* ligation of 10_{SR} and 10b and the subsequent Ni-NTA purification

The solution (from **4.2.13**) containing **10_{SR}** (4.6×10^{-4} mmol, 1.0 equiv.) was adjusted to pH 6.5 and mixed with the solution (6 M Gnd·HCl, 0.2 M phosphate, 20 mM TCEP, pH 6.5, 80 μL) of peptide **10b** (5.5×10^{-4} mmol, 6.9 mM, 1.2 equiv., 9.9 mg). The mixture was degassed by sparging with argon and stirred at 37 °C for 1 h. Subsequently, the crude product **11a** was purified with Ni-NTA resin as stated in general procedures for peptide assembly. The acidic elution was then directly used in the removal of PPH. The yield was calculated by peak-integration of the HPLC profile (λ = 214 nm) (2.1×10^{-4} mmol, 75% HPLC yield).

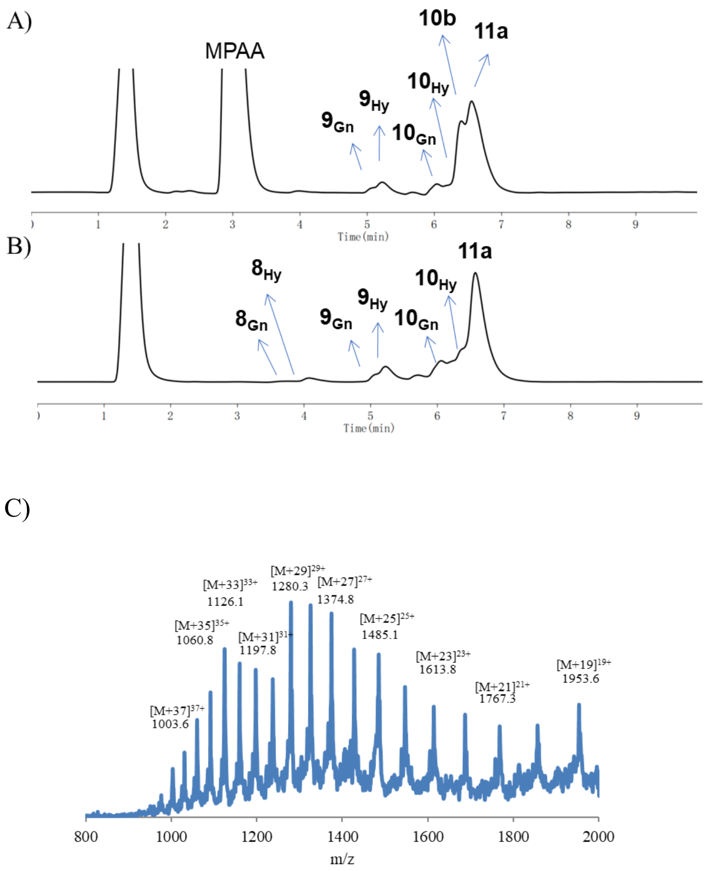

Figure S19. Analytical LCMS trace of **11a**; A) and B): Analytical HPLC of crude and Ni-NTA resin purified **11a**, t_R = 6.6 min (5-30% solvent B in solvent A over 10 min, λ = 214 nm, C4 column); C) ESI-MS calculated for **11a** ($C_{1487}H_{2459}N_{343}O_{730}S_8$): $[M + 19H]^{19+}$ m/z = 1952.5, $[M + 21H]^{21+}$ m/z = 1766.7, $[M + 23H]^{23+}$ m/z = 1613.1, $[M + 25H]^{25+}$ m/z = 1484.2, $[M + 27H]^{27+}$ m/z = 1374.3, $[M + 29H]^{29+}$ m/z = 1279.6, $[M + 31H]^{31+}$ m/z = 1197.1, $[M + 33H]^{33+}$ m/z = 1126.1 $[M + 35H]^{35+}$ m/z = 1060.4, $[M + 37H]^{37+}$ m/z = 1003.1, found: 1953.6, 1767.3, 1613.8, 1485.1, 1374.8, 1280.3, 1197.8, 1126.1, 1060.8, 1003.6.

4.2.15 Removal of PPH to yield 240mer peptide 11b

To the solution (2.5 mL, from **4.2.14**) with eluted **11a** (2.1×10^{-4} mmol) from Ni-NTA resin, the semicarbazide hydrochloride (50 mM, 0.125 mmol, 14 mg) and DTT (50 mM, 0.125 mmol, 19 mg) were added. After degassed with a steady flow of argon for 10 min, the solution was irradiated with UV light (365 nm, 20 W). The reaction was completed in 15 min, and then the product was purified with HPLC and lyophilized to yield a white solid **11b** (3.6 mg, 9.9×10^{-5} mmol, 9% isolated yield from **8a**).

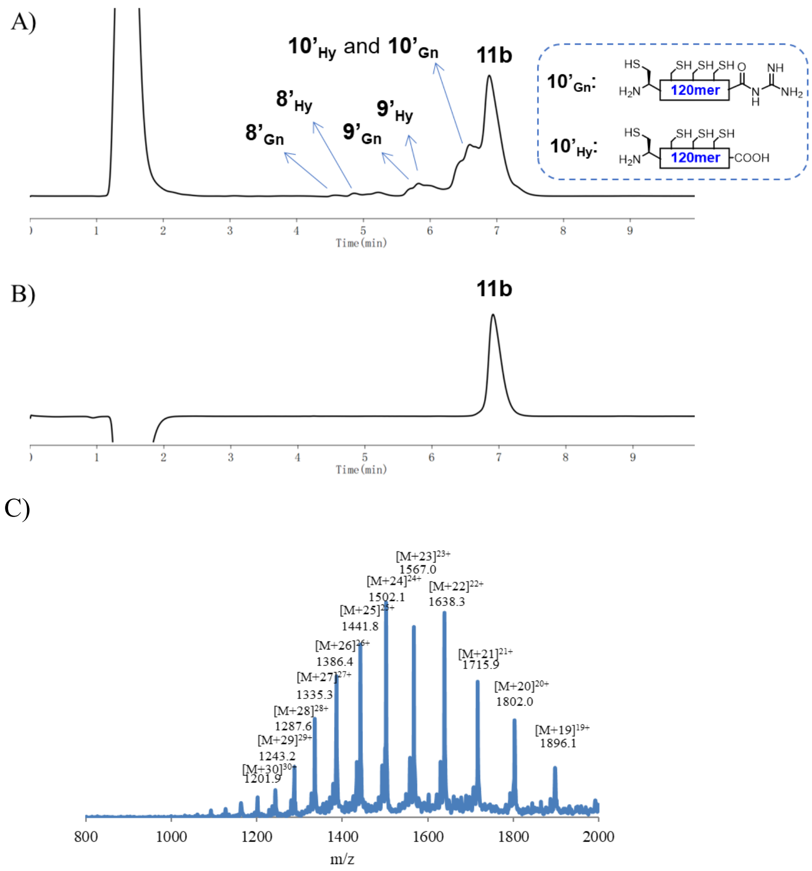

Figure S20. Analytical LCMS trace of the purified **11b**; A) and B): Analytical HPLC of crude and HPLC purified **11b**, t_R = 6.9 min (5-30% solvent B in solvent A over 10 min, λ = 214 nm, C4 column); C) ESI-MS calculated for **11b** ($C_{1440}H_{2404}N_{322}O_{720}S_8$): $[M + 19H]^{19+}$ m/z = 1896.0, $[M + 20H]^{20+}$ m/z = 1801.3, $[M + 21H]^{21+}$ m/z = 1715.5, $[M + 22H]^{22+}$ m/z = 1637.6, $[M + 23H]^{23+}$ m/z = 1566.4, $[M + 24H]^{24+}$ m/z = 1501.2, $[M + 25H]^{25+}$ m/z = 1441.2, $[M + 26H]^{26+}$ m/z = 1385.8, $[M + 27H]^{27+}$ m/z = 1335.3, $[M + 28H]^{28+}$ m/z = 1286.9, $[M + 29H]^{29+}$ m/z = 1242.5, $[M + 30H]^{30+}$ m/z = 1201.1, found: 1896.1, 1802.0, 1715.9, 1638.3, 1567.0, 1502.1, 1441.8, 1386.4, 1335.3, 1287.6, 1243.2, 1201.9.

4.2.16 Synthesis of 10a via ligation of 10_{SR} and 9b and the subsequent Ni-NTA purification

The solution (from **4.2.13**) containing **10_{SR}** (4.6×10^{-4} mmol, 1.0 equiv.) was adjusted to pH 6.5 and mixed with the solution (6 M Gnd·HCl, 0.2 M phosphate, 20 mM TCEP, pH 6.5, 80 μL) of peptide **9b** (5.5×10^{-4} mmol, 6.9 mM, 1.2 equiv., 5.0 mg). The mixture was degassed by sparging with argon and stirred at 37 °C for 1 h. Subsequently, the crude product **12a** was purified with Ni-NTA resin as stated in general procedures for peptide assembly. The acidic elution was then directly used in the removal of PPH. The yield was calculated by peak-integration of the HPLC profile (λ = 214 nm) (2.8×10^{-4} mmol, 61% HPLC yield).

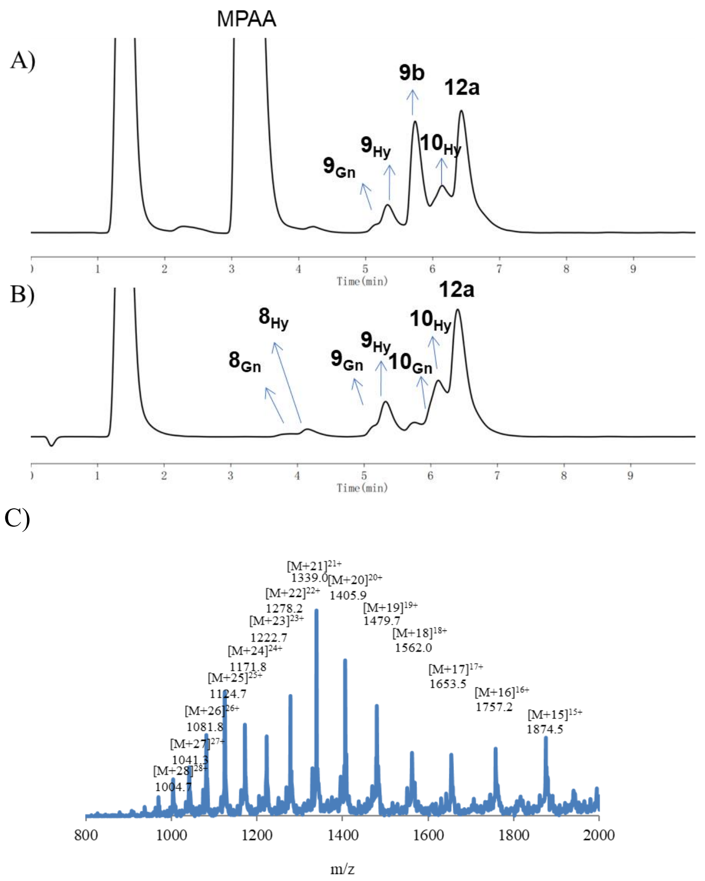

Figure S21. Analytical LCMS trace of **12a**; A) and B): Analytical HPLC of crude and Ni-NTA resin purified **10a**, t_R = 6.4 min (5-30% solvent B in solvent A over 10 min, λ = 214 nm, C4 column) c) ESI-MS calculated for **12a** ($C_{1127}H_{1859}N_{263}O_{550}S_6$): $[M + 15H]^{15+}$ m/z = 1873.4, $[M + 16H]^{16+}$ m/z = 1756.4, $[M + 17H]^{17+}$ m/z = 1653.1, $[M + 18H]^{18+}$ m/z = 1561.3, $[M + 19H]^{19+}$ m/z = 1479.2, $[M + 20H]^{20+}$ m/z = 1405.3, $[M + 21H]^{21+}$ m/z = 1338.4, $[M + 22H]^{22+}$ m/z = 1277.6, $[M + 23H]^{23+}$ m/z = 1222.1, $[M + 24H]^{24+}$ m/z = 1171.2, $[M + 25H]^{25+}$ m/z = 1124.4, $[M + 26H]^{26+}$ m/z = 1081.2, $[M + 27H]^{27+}$ m/z = 1041.2, $[M + 28H]^{28+}$ m/z = 1004.1, found: 1874.5, 1757.2, 1653.5, 1562.0, 1479.7, 1405.9, 1339.0, 1278.2, 1222.7, 1171.8, 1124.7, 1081.8, 1041.3, 1004.7.

4.2.17 Removal of PPH to yield 180mer peptide 12b

To the solution (2.7 mL, from **4.2.16**) with eluted **12a** (2.8×10^{-4} mmol), the semicarbazide hydrochloride (50 mM, 0.135 mmol, 15 mg) and DTT (50 mM, 0.135 mmol, 21 mg) were added. After degassed with a steady flow of argon for 10 min, the solution was irradiated with UV light (365 nm, 20 W). The reaction was complete in 15 min, and then the product was purified with HPLC and lyophilized to yield a white solid **12b** (2.7 mg, 1.0×10^{-4} mmol, 11% isolated yield from **8a**).

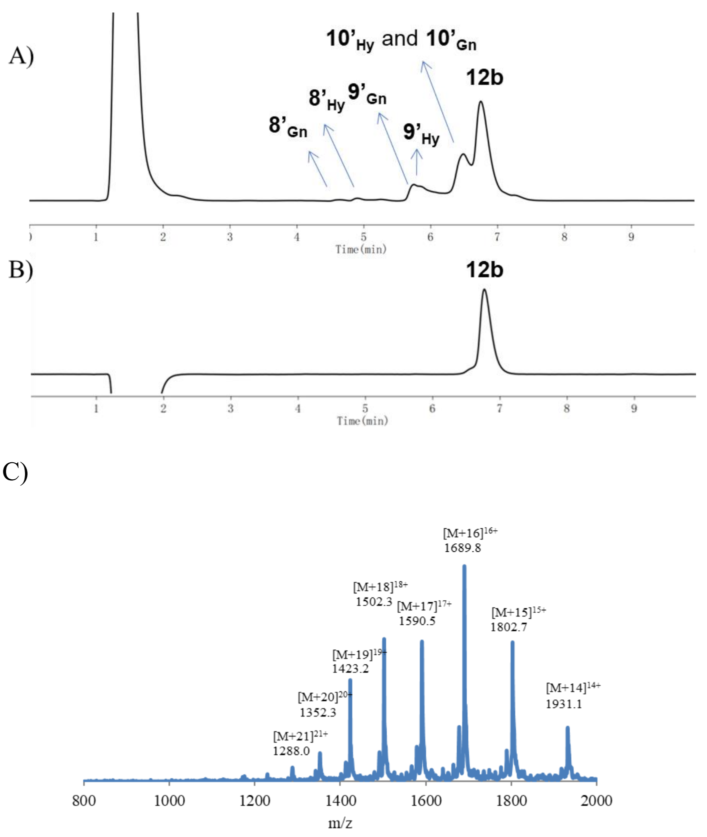

Figure S22. Analytical LCMS trace of the purified **12b**; A) and B): Analytical HPLC of crude and HPLC purified **12b**, t_R = 6.7 min (5-30% solvent B in solvent A over 10 min, λ = 214 nm, C4 column); C) ESI-MS calculated for **12b** ($C_{1080}H_{1804}N_{242}O_{540}S_6$): $[M + 14H]^{14+}$ m/z = 1930.4, $[M + 15H]^{15+}$ m/z = 1801.8, $[M + 16H]^{16+}$ m/z = 1689.2, $[M + 17H]^{17+}$ m/z = 1590.0, $[M + 18H]^{18+}$ m/z = 1501.7, $[M + 19H]^{19+}$ m/z = 1422.7, $[M + 20H]^{20+}$ m/z = 1351.6, $[M + 21H]^{21+}$ m/z = 1287.3, found: 1931.1, 1802.7, 1689.8, 1590.5, 1502.3, 1423.2, 1352.3, 1288.0.

5. Metal-free desulfurization

80mer Muc1 7c:

To a solution (6 M Gnd·HCl, 0.2 M phosphate, 0.3 M TCEP, pH 7.0, 170 μL) of peptide **7b** (0.9 mg, 1.2 × 10^{-5} mmol, 1.0 equiv.), a mixed solution of reduced glutathione (200 mM, 17 μL) and VA-044 (50 mM, 2.0 equiv., 9 μL) in water was added. After degassed with a steady flow of argon for 10 min, the desulfurization was carried out at 65 °C for 3 h, followed by the addition of the mixed solution (26 μL) of reduced glutathione and VA-044 in water every 3 h. After 9 h, the product was purified with HPLC and lyophilized to obtain a white solid **7c** (0.45 mg, 6.0 × 10^{-5} mmol, 50% isolated yield)

A)

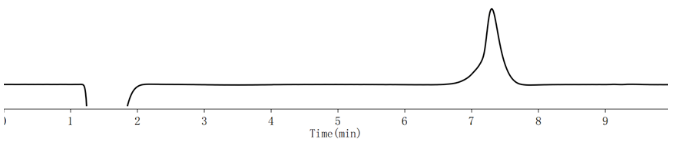

B)

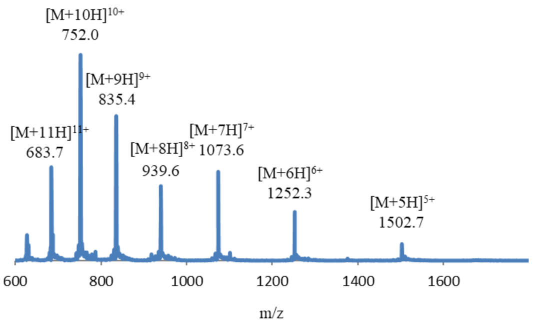

Figure S23. Analytical LCMS trace of the purified **7c**; A) Analytical HPLC: t_R = 7.2 min (2-30% solvent B in solvent A over 10 min, λ = 214 nm, C4 column); B) ESI-MS calculated for **7c** ($C_{320}H_{504}N_{102}O_{108}$): $[M + 5H]^{5+}$ m/z = 1502.6, $[M + 6H]^{6+}$ m/z = 1252.4, $[M + 7H]^{7+}$ m/z = 1073.6, $[M + 8H]^{8+}$ m/z = 939.5, $[M + 9H]^{9+}$ m/z = 835.2, $[M + 10H]^{10+}$ m/z = 751.8, $[M + 11H]^{11+}$ m/z = 683.6, found: 1502.7, 1252.3, 1073.6, 939.6, 835.4, 752.0, 683.7.

30mer AFGP mimic 8c:

To a solution (6 M Gnd·HCl, 0.2 M phosphate, 0.3 M TCEP, pH 7.0, 923 μL) of peptide **8b** (10mg, 2.2 × 10^{-3} mmol, 1 equiv.), a mixed solution of reduced glutathione (200 mM, 183 μL) and VA-044 (50 mM, 2.0 equiv., 44 μL) in water was added. After degassed with a steady flow of argon for 10 min, the desulfurization was carried out at 65 °C for 3 h, followed by the addition of the mixed solution (450 μL) of reduced glutathione and VA-044 in water every 3 h. After 9 h, the product was purified with HPLC and lyophilized to obtain a white solid **8c** (4.7 mg, 1.0 × 10^{-3} mmol, 45% isolated yield)

A)

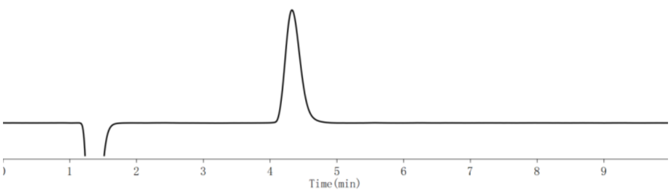

B)

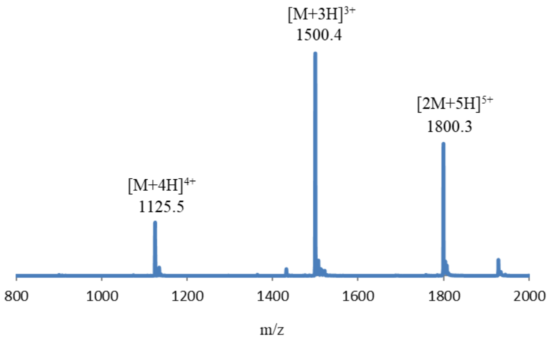

Figure S24. Analytical LCMS trace of the purified **8c**; A) Analytical HPLC: $t_R = 4.4$ min (5-30% solvent B in solvent A over 10 min, $\lambda = 214$ nm, C4 column); B) ESI-MS calculated for **8c** ($C_{180}H_{304}N_{42}O_{90}$): $[2M + 5H]^{5+}$ m/z = 1799.8, $[M + 3H]^{3+}$ m/z = 1500.0, $[M + 4H]^{4+}$ m/z = 1125.2, found: 1800.3, 1500.4, 1125.5.

60mer AFGP mimic 9c:

To a solution (6 M Gnd·HCl, 0.2 M phosphate, 0.3 M TCEP, pH 7.0, 1 mL) of peptide **9b** (5mg, 5.5×10^{-4} mmol, 1.0 equiv.), a mixed solution of reduced glutathione (200 mM, 100 µL) and VA-044 (50 mM, 2.0 equiv., 22 µL) in water was added. After degassed with a steady flow of argon for 10 min, the desulfurization was carried out at 65 °C for 3 h, followed by the addition of the mixed solution (500 µL) of reduced glutathione and VA-044 in water every 3 h. After 9 h, the product was purified with HPLC and lyophilized to obtain a white solid **9c** (2.5 mg, 2.8×10^{-4} mmol, 50% isolated yield).

A)

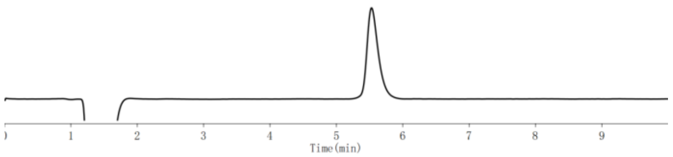

B)

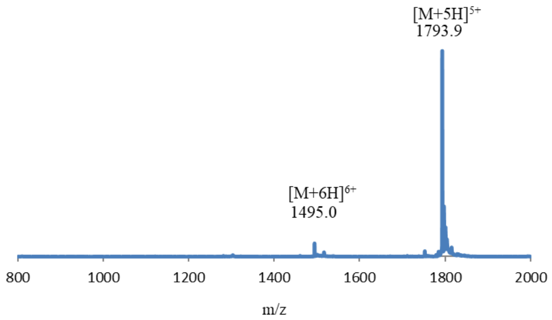

Figure S25. Analytical LCMS trace of the purified **9c**; A) Analytical HPLC: t_R = 5.7 min (5-30% solvent B in solvent A over 10 min, λ = 214 nm, C4 column); B) ESI-MS calculated for **9c** ($C_{360}H_{604}N_{82}O_{180}$): $[M + 5H]^{5+}$ m/z = 1793.2, $[M + 6H]^{6+}$ m/z = 1494.5, found: 1793.9, 1495.0.

120mer AFGP mimic 10c:

To a solution (6 M Gnd·HCl, 0.2 M phosphate, 0.3 M TCEP, pH 7.0, 800 μL) of peptide **10b** (4mg, 2.2 × 10^{-4} mmol, 1.0 equiv.), a mixed solution of reduced glutathione (200 mM, 80 μL) and VA-044 (50 mM, 2.0 equiv., 18 μL) in water was added. After degassing with a steady flow of argon for 10 min, the desulfurization was carried out at 65 °C for 3 h, followed by the addition of the mixed solution (400 μL) of reduced glutathione and VA-044 in water every 3 h. After 9 h, the product was purified with HPLC and lyophilized to obtain a white solid **10c** (2.3 mg, 1.3 × 10^{-4} mmol, 58% isolated yield)

A)

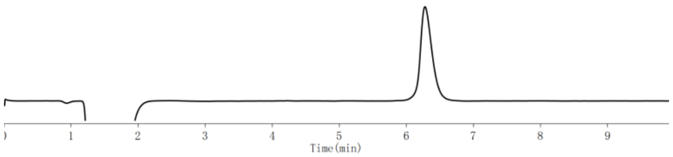

B)

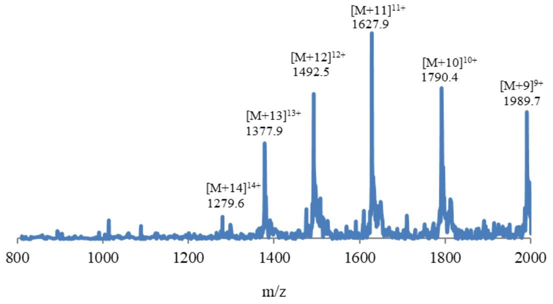

Figure S26. Analytical LCMS trace of the purified **10c**; A) Analytical HPLC: t_R = 6.2 min (5-30% solvent B in solvent A over 10 min, λ = 214 nm, C4 column); B) ESI-MS calculated for **10c** ($C_{720}H_{1204}N_{162}O_{360}$): $[M + 9H]^{9+}$ m/z = 1989.0, $[M + 10H]^{10+}$ m/z = 1790.0, $[M + 11H]^{11+}$ m/z = 1627.4, $[M + 12H]^{12+}$ m/z = 1491.9, $[M + 13H]^{13+}$ m/z = 1377.2, $[M + 14H]^{14+}$ m/z = 1278.9, found: 1989.7, 1790.4, 1627.9, 1492.5, 1377.9, 1279.6.

240mer AFGP mimic 11c:

To a solution (6 M Gnd·HCl, 0.2 M phosphate, 0.3 M TCEP, pH 7.0, 500 μL) of peptide **11b** (2.5mg, 7.0 × 10^{-5} mmol, 1.0 equiv.), a mixed solution of reduced glutathione (200 mM, 50 μL) and VA-044 (50 mM, 2.0 equiv., 11 μL) in water was added. After degassing with a steady flow of argon for 10 min, the desulfurization was carried out at 65 °C for 3 h, followed by the addition of the mixed solution (250 μL) of reduced glutathione and VA-044 in water every 3 h. After 9 h, the product was purified with HPLC and lyophilized to obtain a white solid **11c** (1.8 mg, 4.9 × 10^{-5} mmol, 70% isolated yield)

A)

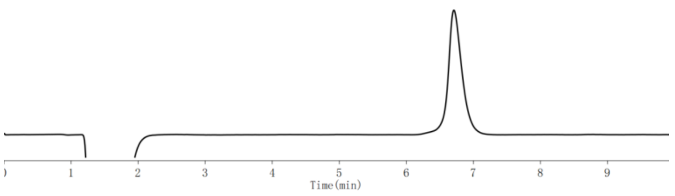

B)

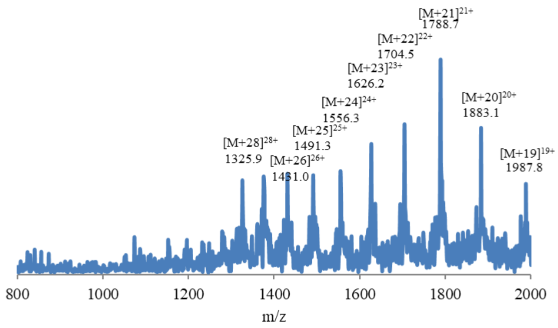

Figure S27. Analytical LCMS trace of the purified **11c**; A) Analytical HPLC: t_R = 6.8 min (5-30% solvent B in solvent A over 10 min, λ = 214 nm, C4 column); B) ESI-MS calculated for **11c** ($C_{1440}H_{2404}N_{322}O_{720}$): $[M + 19H]^{19+}$ m/z = 1987.0, $[M + 20H]^{20+}$ m/z = 1882.5, $[M + 21H]^{21+}$ m/z = 1788.4, $[M + 22H]^{22+}$ m/z = 1703.3, $[M + 23H]^{23+}$ m/z = 1625.9, $[M + 24H]^{24+}$ m/z = 1555.3, $[M + 25H]^{25+}$ m/z = 1490.5, $[M + 26H]^{26+}$ m/z = 1430.9, $[M + 28H]^{28+}$ m/z = 1325.0, found: 1987.8, 1883.1, 1788.7, 1704.5, 1626.2, 1556.3, 1491.3, 1431.0, 1325.9.

180mer AFGP mimic 12c:

To a solution (6 M Gnd·HCl, 0.2 M phosphate, 0.3 M TCEP, pH 7.0, 800 μL) of peptide **12b** (2.7 mg, 1.0×10^{-4} mmol, 1.0 equiv.), a mixed solution of reduced glutathione (200 mM, 80 μL) and VA-044 (50 mM, 2.0 equiv., 18 μL) in water was added. After degassing with a steady flow of argon for 10 min, the desulfurization was carried out at 65 °C for 3 h, followed by the addition of the mixed solution (400 μL) of reduced glutathione and VA-044 in water every 3 h. After 9 h, the product was purified with HPLC and lyophilized to obtain a white solid **12c** (1.5 mg, 5.7×10^{-5} mmol, 57% isolated yield)

A)

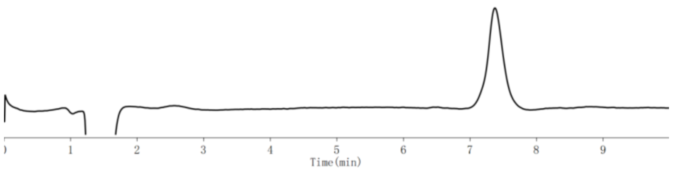

B)

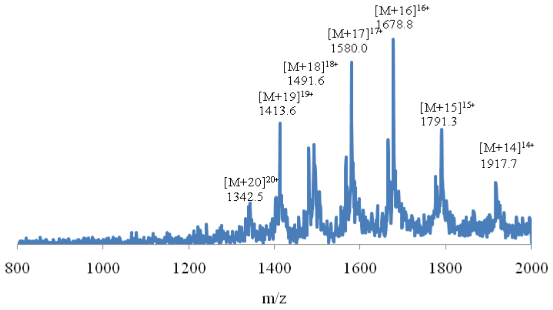

Figure S28. Analytical LCMS trace of the purified **12c**; A) Analytical HPLC: t_R = 7.4 min (5-30% solvent B in solvent A over 10 min, λ = 214 nm, C4 column); B) ESI-MS calculated for **12c** ($C_{1080}H_{1804}N_{242}O_{540}$): $[M + 14H]^{14+}$ m/z = 1916.7, $[M + 15H]^{15+}$ m/z = 1789.0, $[M + 16H]^{16+}$ m/z = 1677.2, $[M + 17H]^{17+}$ m/z = 1578.6, $[M + 18H]^{18+}$ m/z = 1491.0, $[M + 19H]^{19+}$ m/z = 1412.6, $[M + 20H]^{20+}$ m/z = 1342.0, found: 1917.7, 1791.3, 1678.8, 1580.0, 1491.6, 1413.6, 1342.5.

6. Circular dichroism spectropolarimetry

All synthetic AFGP mimics were dissolved in Milli-Q water (0.1 mg/mL) and incubated at 0, 10, 20, 30 and 40 °C. Far-UV CD spectra were carried out by means of Jasco J-815 Spectropolarimeter[7] with cell length of 1 mm and the scan wavelength of from 190 nm to 240 nm.

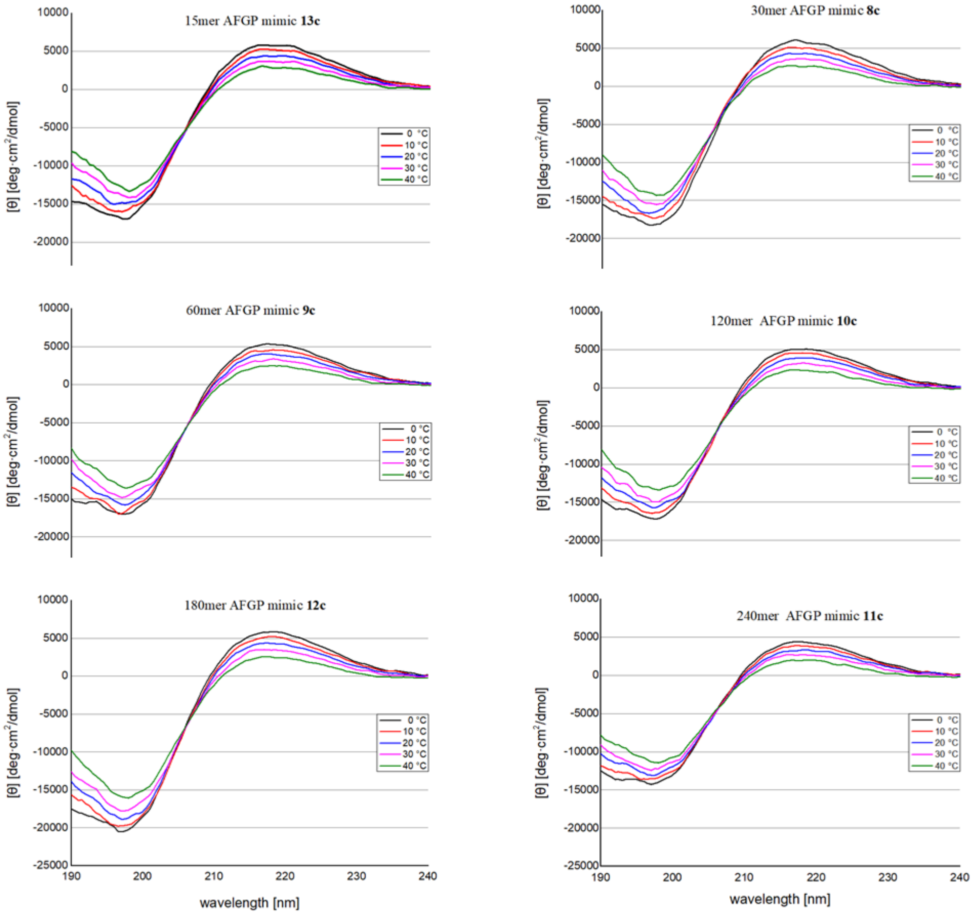

Figure S29. Circular dichroism spectra of synthetic AFGP mimic **13c**, **8c**, **9c**, **10c**, **12c**, **11c** at various temperature ranging from 0 °C to 40 °C.

7. Evaluation of antifreeze activity

The measurement of thermal hysteresis activity (THA) to evaluate antifreeze activity was performed by means of Otago Nanoliter Osmometer (Otago Osmometers Ltd., Dunedin, New Zealand) according to the procedures reported previously[8]. TH (TH = |Tm-Tf|) is defined to be the difference between melting point and freezing point of AF(G)P solution.

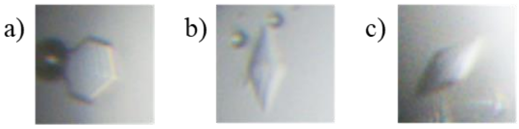

Figure S30. Ice crystal morphology in the presence of synthetic AFGP mimic at 10 mg/mL: a) 15mer AFGP mimic **13c**, b) 30mer AFGP mimic **8c**, c) 60mer AFGP mimic **9c**.

8. NMR-spectra of novel compounds

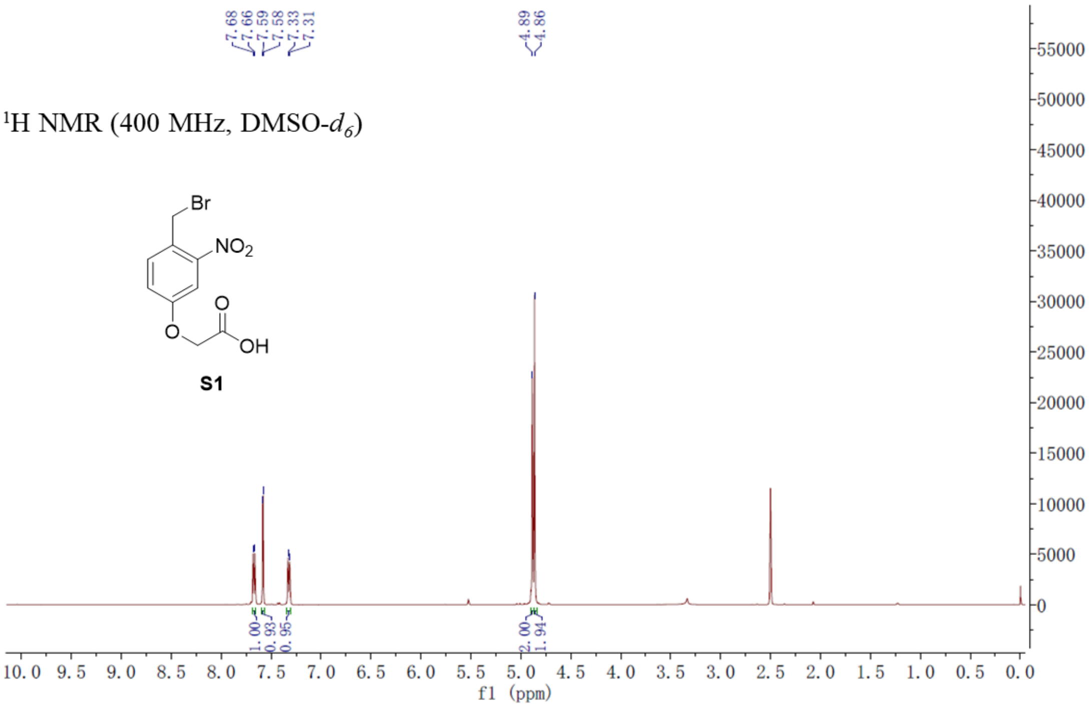

Figure S31. ^{1}H NMR spectrum of compound **S1**.

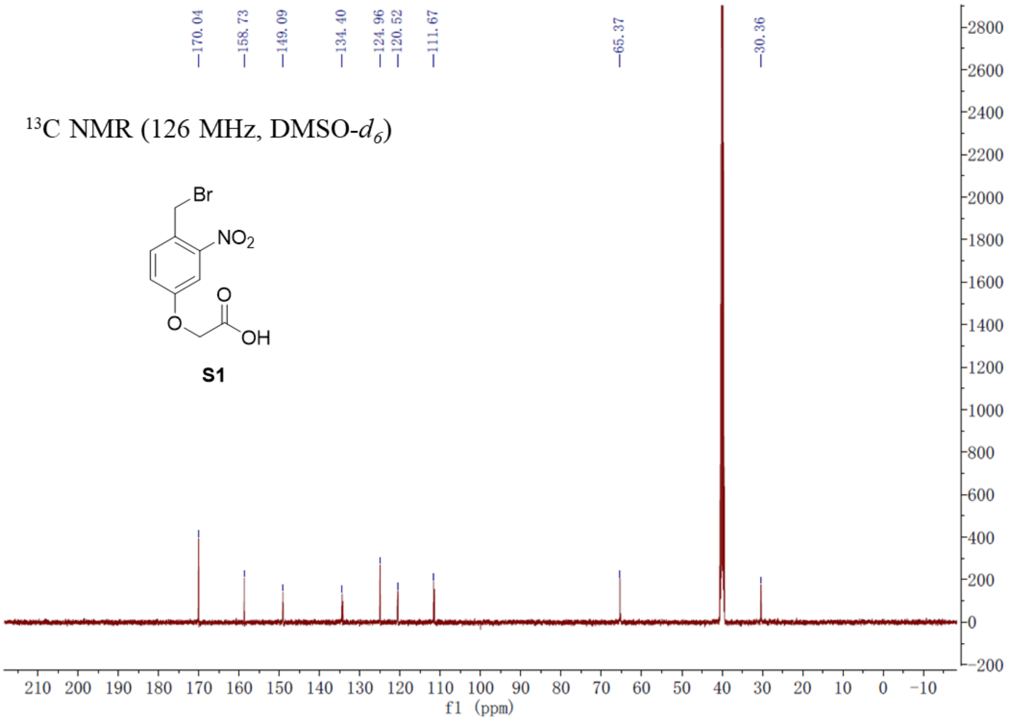

Figure S32. ^{13}C NMR spectrum of compound **S1**.

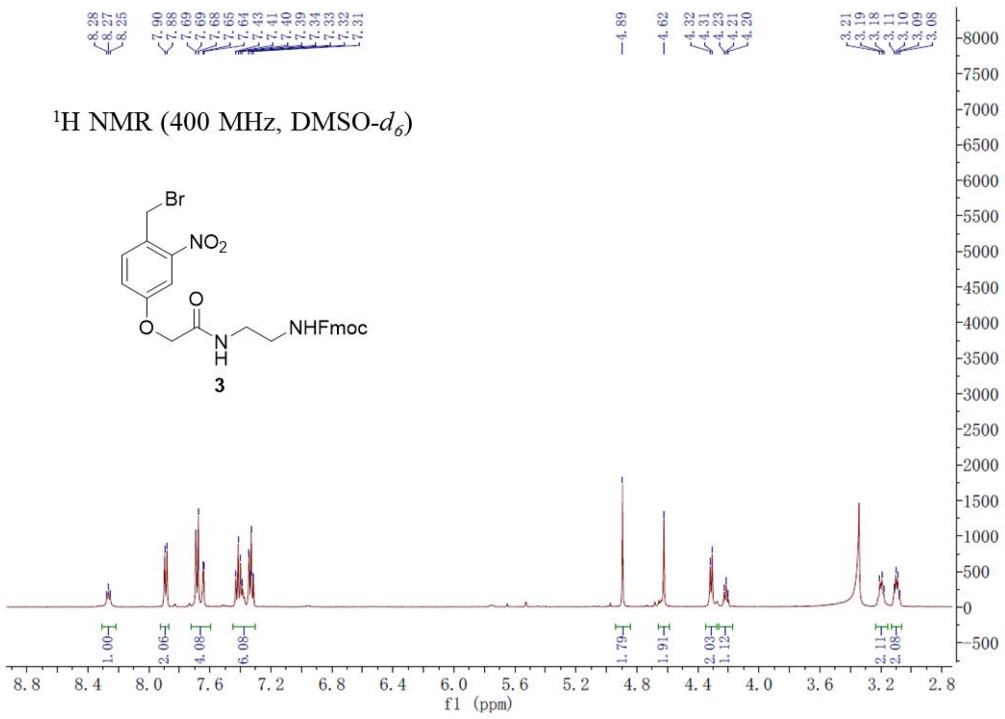

Figure S33. ^1H NMR spectrum of compound **3.**

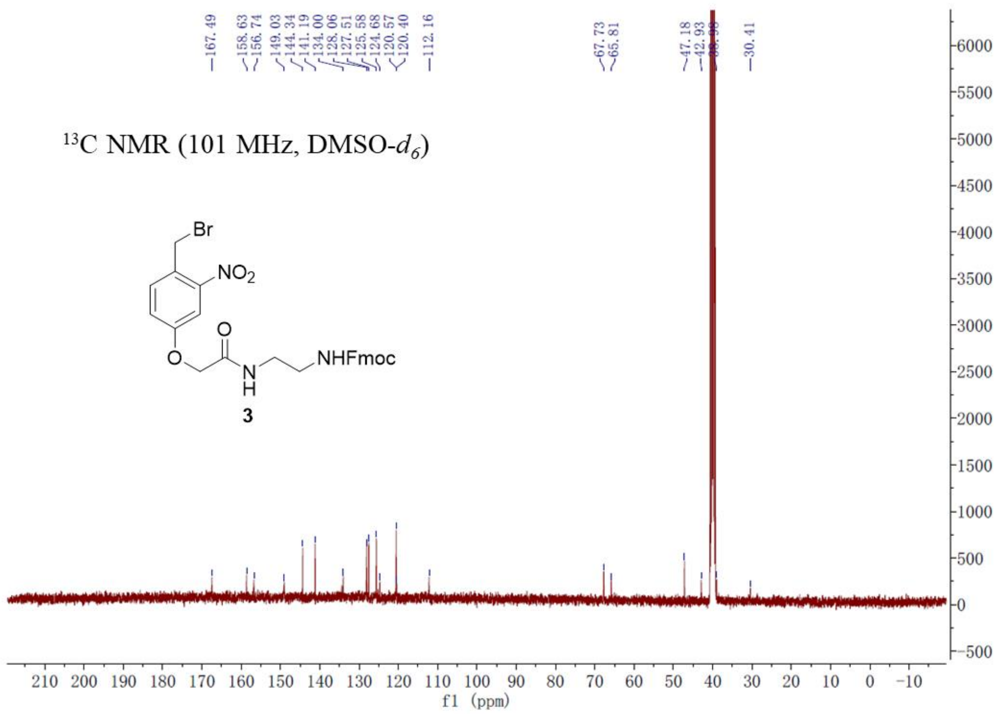

Figure S34. ^{13}C NMR spectrum of compound **3**

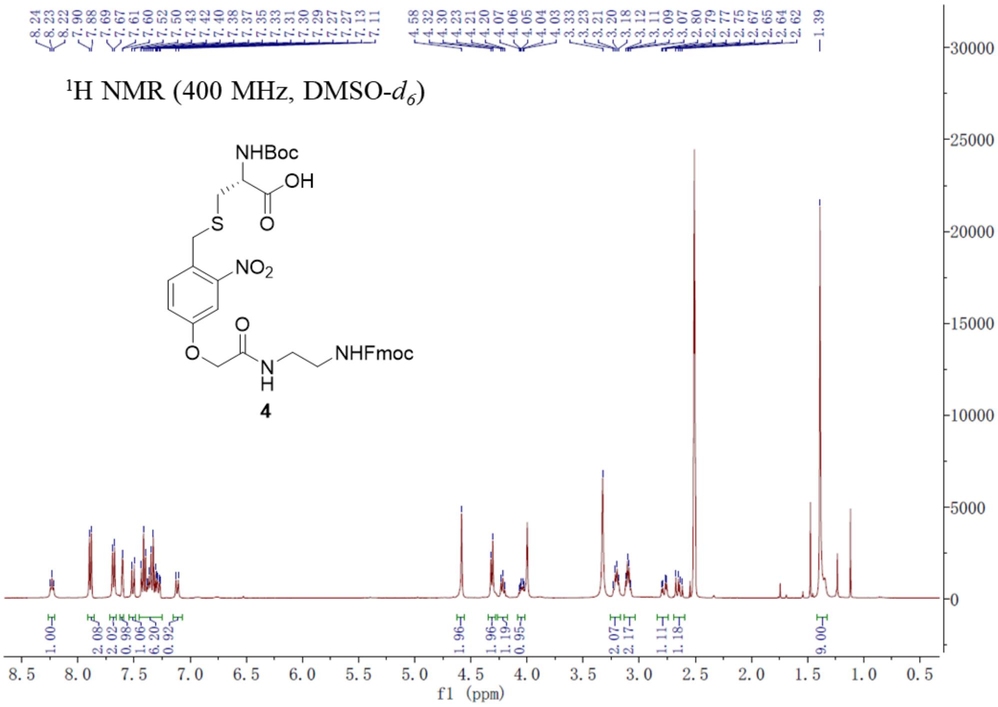

Figure S35. ^{1}H NMR spectrum of compound **4**.

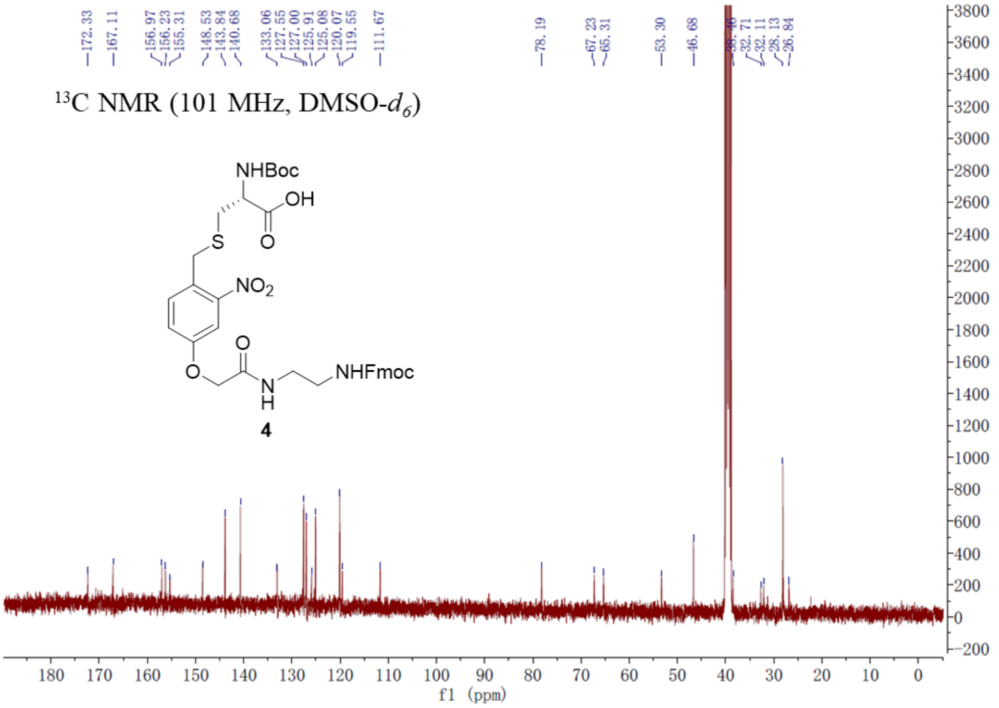

Figure S36. ^{13}C NMR spectrum of compound **4**.

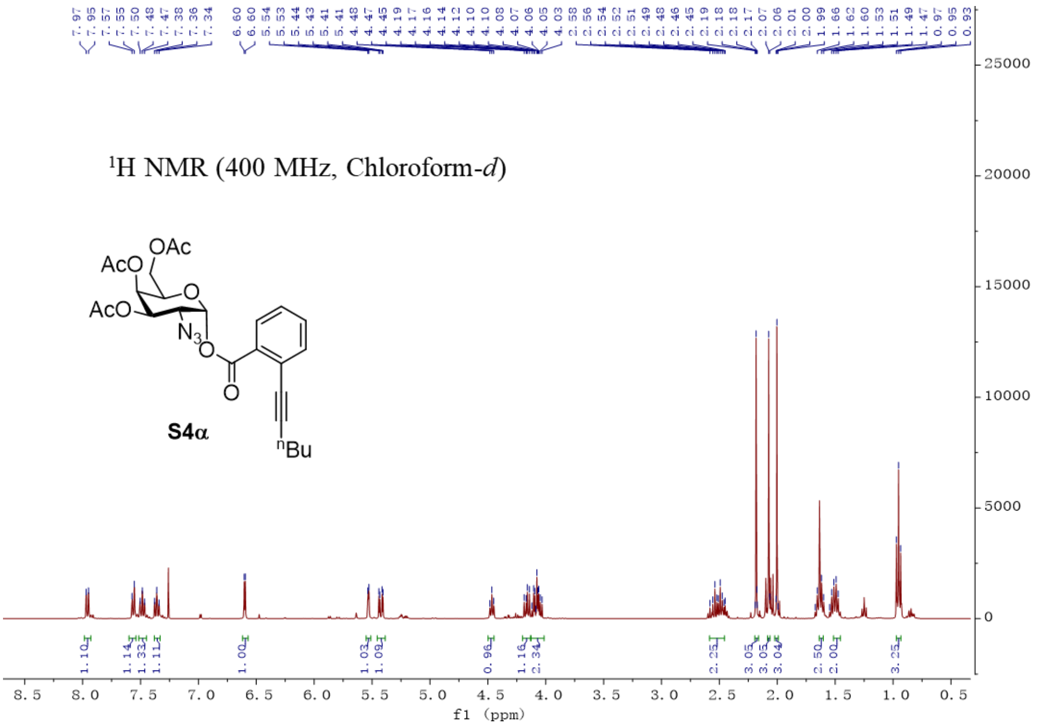

Figure S37. ^{1}H NMR spectrum of compound **S4α**.

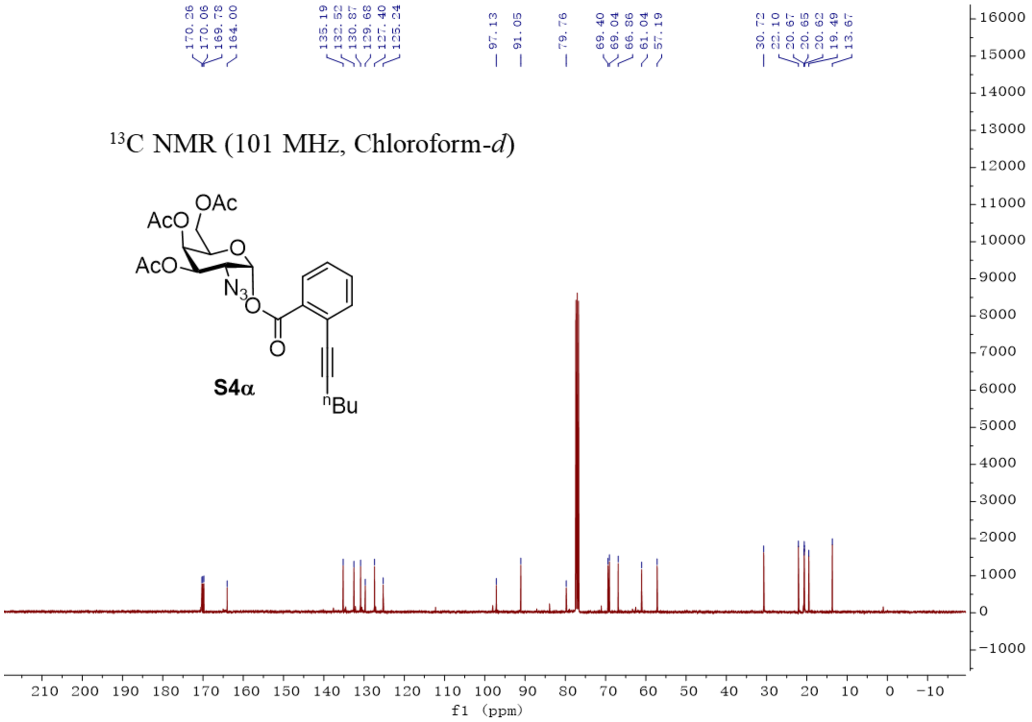

Figure S38. ^{13}C NMR spectrum of compound **S4α**.

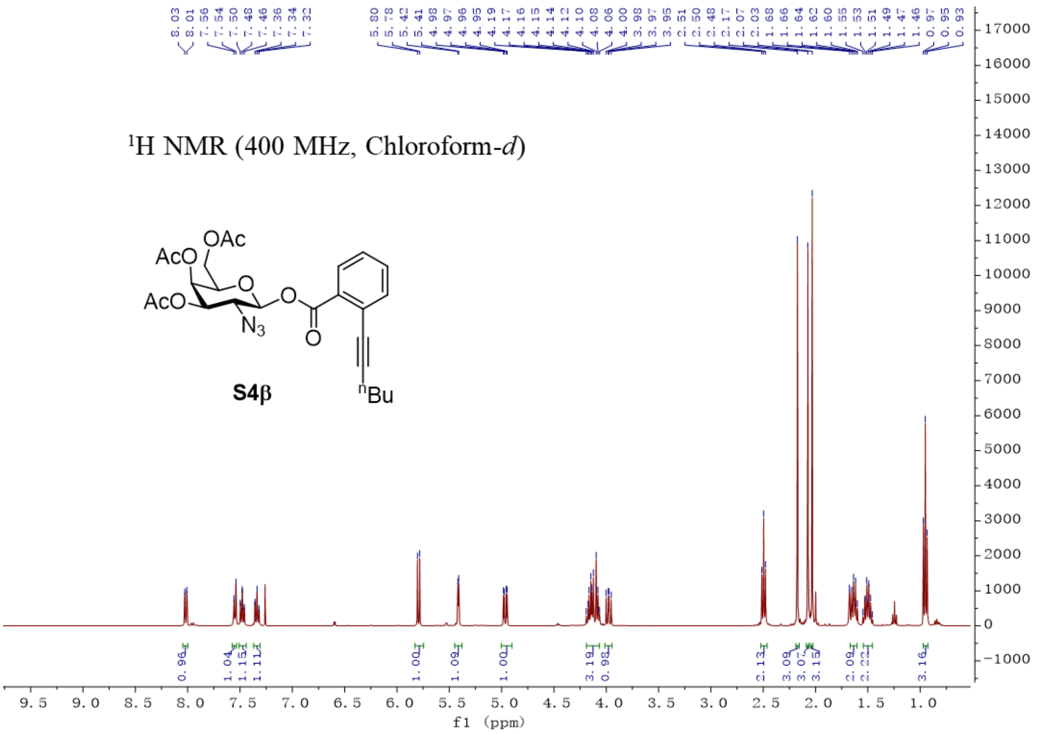

Figure S39. ^{1}H NMR spectrum of compound **S4β**.

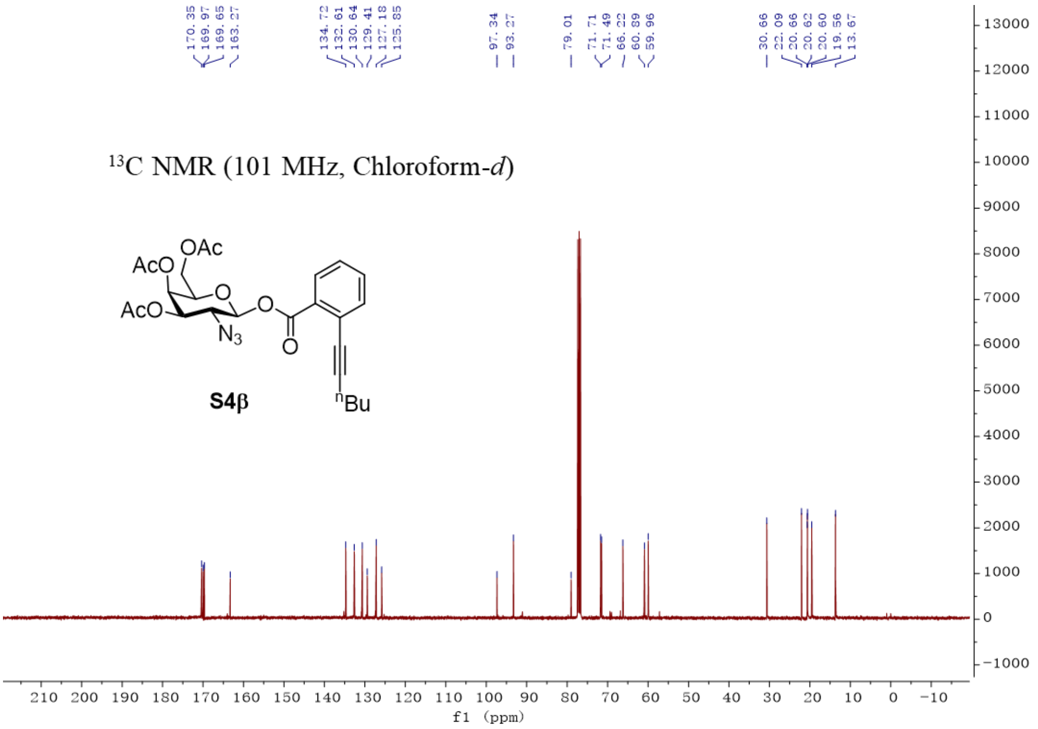

Figure S40. ^{13}C NMR spectrum of compound **S4β**.

9. High-resolution mass spectra of AFGP mimic **10c**, **12c**, **11c**.

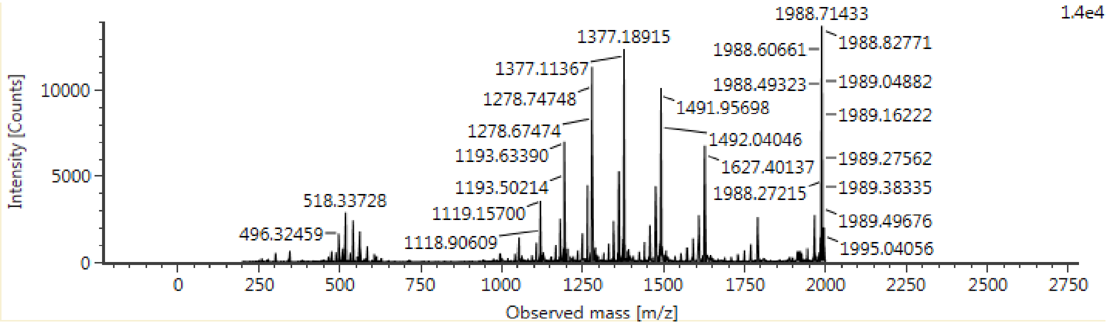

Figure S41. HRMS (ESI) of antifreeze segment **10c**. Molecular mass (Average): calculated for $C_{720}H_{1204}N_{162}O_{360}$: 17881.09587 Da; found: 17881.28700 Da, error: 10.689 ppm.

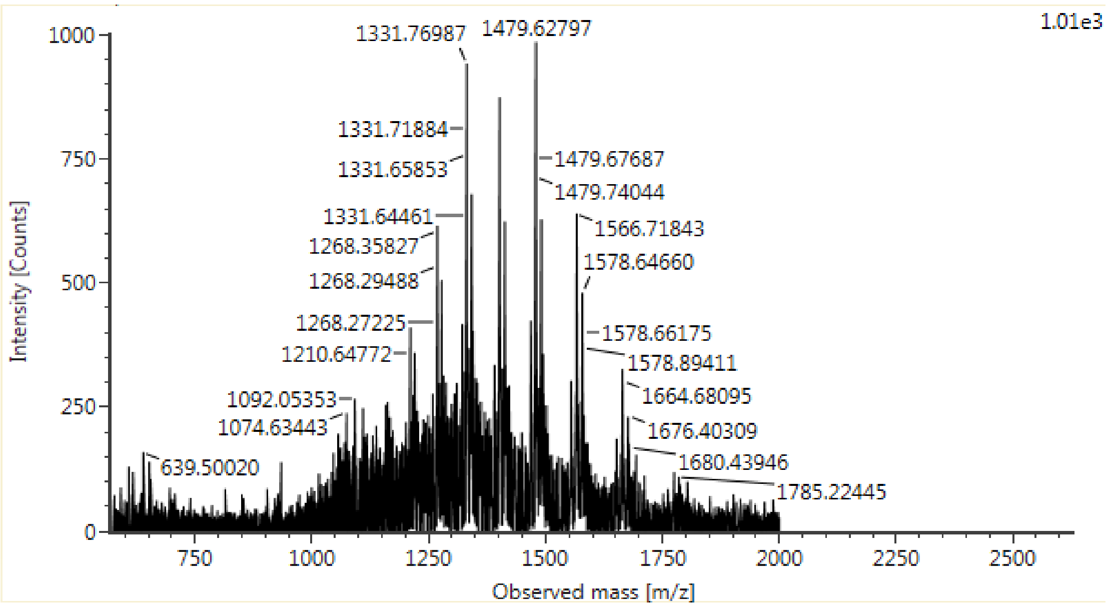

Figure S42. HRMS (ESI) of antifreeze segment **12c**. Molecular mass (Average): calculated for $C_{1080}H_{1804}N_{242}O_{540}$: 26819.46600 Da; found: 26819.00000 Da, error: 17.380 ppm.

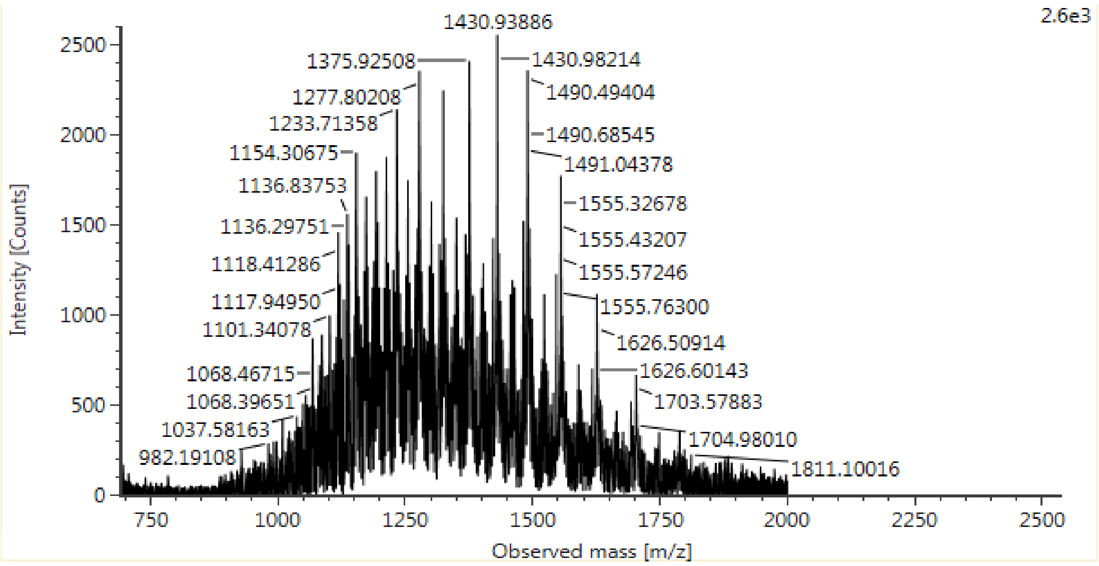

Figure S43. HRMS (ESI) of antifreeze segment **11c**. Molecular mass (Average): calculated for $C_{1440}H_{2404}N_{322}O_{720}$: 35748.60600 Da; found: 35748.00000 Da, error: 16.950 ppm.

10. Reference

1. M. S. Baker, Y. Vinita, S. Ayusman and S. T. Phillips, *Angew. Chem. Int. Ed.*, 2013, **52**, 10295.
2. M. Simone, K. Janos, A. Nahid, W. Stefanie, T. Alexander, P. H. Seeberger, G. Brezesinski and L. Hartmann, *Biomacromolecules*, 2014, **15**, 1687.
3. K. M. Koeller, M. E. B. Smith and C.-H. Wong, *Bioorg. Med. Chem.*, 2000, **8**, 1017.
4. T. Tang, J. Li, Y. Zhu, Y. Li and B. Yu, *J. Am. Chem. Soc.*, 2013, **135**, 18396.
5. Y.-L. Zhang, S. M. Muthana, J. J. Barchi and J. C. Gildersleeve, *Org. Lett.*, 2012, **14**, 3958.
6. H. Huang and D. L. Rabenstein, *J. Peptide Res.*, 1999, **53**, 548.
7. C. A. Bush and R. E. Feeney, *Chem. Biol. Drug Des.*, 2010, **28**, 386.
8. M. Takamichi, Y. Nishimiya, A. Miura and S. Tsuda, *FEBS J.*, 2010, **274**, 6469.
